# Structures of the mumps virus polymerase complex via cryo-electron microscopy

**DOI:** 10.1038/s41467-024-48389-9

**Published:** 2024-05-17

**Authors:** Tianhao Li, Mingdong Liu, Zhanxi Gu, Xin Su, Yunhui Liu, Jinzhong Lin, Yu Zhang, Qing-Tao Shen

**Affiliations:** 1 School of Life Sciences, Department of Chemical Biology, Southern University of Science and Technology, Shenzhen, 518055 China; 2 Laboratory for Marine Biology and Biotechnology, Qingdao National Laboratory for Marine Science and Technology, Qingdao, 266237 China; 3 Institute for Biological Electron Microscopy, Southern University of Science and Technology, Shenzhen, 518055 China; 4 School of Life Science and Technology, ShanghaiTech University, Shanghai, 201210 China; 5 University of Chinese Academy of Sciences, Beijing, 100049 China; 6 Key Laboratory of Synthetic Biology, Center for Excellence in Molecular Plant Sciences, Institute of Plant Physiology and Ecology, Chinese Academy of Sciences, Shanghai, 200032 China; 7 State Key Laboratory of Genetic Engineering, School of Life Sciences, Zhongshan Hospital, Fudan University, Shanghai, 200438 China

**Keywords:** Cryoelectron microscopy, Virus structures

## Abstract

The viral polymerase complex, comprising the large protein (L) and phosphoprotein (P), is crucial for both genome replication and transcription in non-segmented negative-strand RNA viruses (nsNSVs), while structures corresponding to these activities remain obscure. Here, we resolved two L–P complex conformations from the mumps virus (MuV), a typical member of nsNSVs, via cryogenic-electron microscopy. One conformation presents all five domains of L forming a continuous RNA tunnel to the methyltransferase domain (MTase), preferably as a transcription state. The other conformation has the appendage averaged out, which is inaccessible to MTase. In both conformations, parallel P tetramers are revealed around MuV L, which, together with structures of other nsNSVs, demonstrates the diverse origins of the L-binding X domain of P. Our study links varying structures of nsNSV polymerase complexes with genome replication and transcription and points to a sliding model for polymerase complexes to advance along the RNA templates.

## Abstract (web summary)

The viral polymerase complex is crucial for both genome replication and transcription in non-segmented negative-strand RNA viruses. Here, the authors link varying structures of polymerase complexes with their dual functions and propose a sliding model for them to advance along the RNA templates.

## Introduction

The non-segmented negative-strand RNA viruses (nsNSVs) contain many pathogens, including the Ebola virus (EBOV), rabies virus (RABV), human respiratory syncytial virus (HRSV), and mumps virus (MuV), which cause severe human disease and even death^1,2^. During the whole viral life cycle, viral genomes are always enwrapped by viral nucleoproteins (N), forming the helical nucleocapsids (NC) for genome protection and encapsulation^3–5^. After viral entry into host cells, another two viral proteins, including large proteins (L) and phosphoproteins (P), along with NC, are released into cytosols^6^. L and P function as the RNA polymerase complex, responsible for replicating and transcribing the viral genome^7–12^.

To catalyze the RNA synthesis in both replication and transcription, L sequentially consists of five domains: the RNA-dependent RNA polymerase domain (RdRp), polyribonucleotidyl transferase domain (PRNTase), connector domain (CD), methyltransferase domain (MTase), and C-terminal domain (CTD)^13–15^. As the core module of L, RdRp and PRNTase take charge of the RNA synthesis and capping^8,16,17^. MTase has methylation activity and is only required for transcription^11,18–20^. RdRp-PRNTase is quite conserved in structure amongst nsNSVs, while CD-MTase-CTD resembles an appendage of RdRp-PRNTase with great structural diversity^15,21–26^. Specifically, CD-MTase-CTD from HRSV, human metapneumovirus (HMPV), and EBOV are not resolved in structures due to the inherent flexbilities^21,22,24,26^, and show distinct spatial organizations in vesicular stomatitis Indiana virus (VSIV), RABV, and parainfluenza virus 5 (PIV-5)^15,23,25,27^. Limited by the number of complete L structures and the lack of functional analyses, the relationship between these conformations and RNA synthesis remains elusive.

P is the polymerase cofactor of L for RNA synthesis^28–33^. P harbors an oligomerization domain (P_OD_) for self-oligomerization, and the oligomeric P attaches to RdRp of L, tethering the polymerase to NC to extract the RNA strand for both replication and transcription^34–38^. All resolved L–P complexes in HRSV, PIV-5, and EBOV reveal four parallel P molecules^21–23,26^. Immediately after P_OD_, there is an X domain (P_XD_) within the C-terminal domain (P_CTD_), which can bind both RdRp and N^23,39^. Remarkably, the P_XD_-binding sites of N show diversities in nsNSVs, from the RNA-binding domain N_CORE_ to the molecular recognition element (MoRE) motif within the C-terminus of N (N_TAIL_)^36,37,39–45^. Since four P_OD_ assemble into a coiled-coil structure, a model was proposed that P tetramer cartwheels on NC during the advance of the polymerase^46–49^. Once the P_OD_ rotates, the L-anchored P_XD_ is assumed to dissociate from RdRp, and P_XD_ from another P will rebind L. The cartwheeling model requires the binding capability of all four P_XD_ for the iterative cycles. However, recent studies demonstrated that P tetramers with one to three impaired P_XD_ still maintain a comparable or even higher bioactivity of RNA synthesis^39^. Even surprisingly, only one N binding-competent P_XD_ in the tetramer is enough for the minigenome transcription^39,50^. All these reach another sliding model that does not require the oligomeric P to undergo rotation. Unfortunately, both models still lack structural evidence, leaving the L–P advance on NC obscure.

The mumps virus, which belongs to the family *Paramyxoviridae*, is a typical member of nsNSVs that causes acute upper respiratory symptoms and parotitis. Despite available vaccines, several regional outbreaks have still occurred worldwide in the past decades^51–53^. Previous studies on MuV N and P indicated some unique mechanisms that are inconsistent with other well-studied species^37,40,54^. P_CTD_ was identified as the sole L-binding region in MuV, while P_CTD_ alone could not form a stable complex with L in the absence of P_OD_^41^. More surprisingly, the recombinant MuV P_OD_ prefers the formation of anti-parallel tetramers (parallel dimers in anti-parallel configuration)^36,41^. These unusual findings on MuV P and L need further verification on the L–P complex, which will enrich the structure pool of polymerase complexes and benefit the comprehensive understanding of the molecular mechanism for replication and transcription.

Here, we resolved two MuV L–P complex conformations via cryogenic-electron microscopy (cryo-EM). One conformation presents all five domains of L, among which CD-MTase-CTD adopts a spatial organization distinct from PIV-5, with a continuous RNA tunnel from RdRp-PRNTase to CD-MTase-CTD, preferably as a transcription state. The other conformation has CD-MTase-CTD averaged out due to the structural flexibility, with its RNA tunnel inaccessible to MTase, unfavorable for genome transcription. Moreover, parallel P tetramers are revealed in MuV L–P complexes, and our atomic model of MuV P helps in building uncertain residues to the C-terminal regions at the front of the X domain of P (P_XD_) in PIV-5, which, together with other structures, demonstrates the diverse origins of L-binding P_XD_ from the P tetramer in nsNSVs.

## Results

### Two conformations of MuV L–P complex

We co-expressed MuV L and P in Sf9 cells. The recombinant proteins were purified by tandem Strep-Tactin affinity, ion exchange, and size-exclusion chromatography (Supplementary Fig. 1a). SDS-PAGE and western blot analyses verified the assembly of MuV L–P complex from full-length individuals (Supplementary Fig. 1b,c), and the de novo RNA synthesis assay further showed the catalytic activity of MuV L–P as the RNA-dependent RNA polymerase (Supplementary Fig. 1d,e). To unveil the architecture of MuV L–P complex, purified proteins were subjected to cryo-EM analyses. Three-dimensional (3D) classification and refinements revealed two distinct conformations: one resembles the density map of VSIV L, with both the body and the appendage visible (termed L_integral_–P); the other only has the body of L (termed L_body_–P), similar to HRSV, HMPV and EBOV^21,22,24,26^ (Supplementary Fig. 2). The missing of appendage in L_body_–P indicates the structural flexibility in MuV L–P, as in other nsNSVs.

**Fig. 1:**
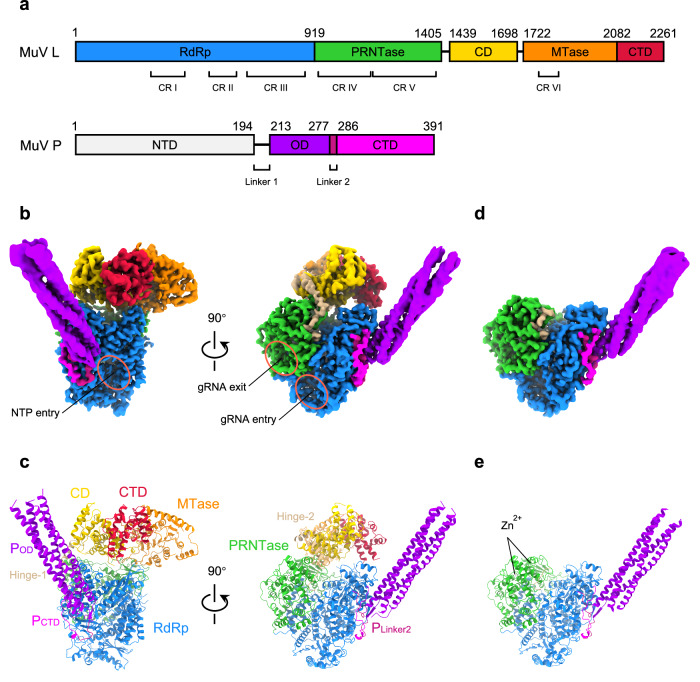
Structures of MuV L–P complex. **a** Diagram of MuV L and P domains. RdRp, PRNTase, Hinge regions (Hinge-1&2), CD, MTase, and CTD of L are colored in blue, green, tan, gold, orange, and crimson, respectively. NTD, OD, Linker region 2 (Linker2), and CTD of P are colored in light gray, purple, violet red, and magenta, respectively. The same color strategy is used throughout the manuscript unless specified. CR I–VI: six conserved regions in L. **b** Cryo-EM density maps of MuV L_integral_–P (EMD-35864). NTP entry, genomic RNA (gRNA) entry, and gRNA exit are circled. **c** Atomic models of MuV L_integral_–P (PDB ID 8IZL). **d** Cryo-EM density map of MuV L_body_–P (EMD-37964). **e** Atomic model of MuV L_body_–P (PDB ID 8X01). Maps in (**b**, **d**) are the composite cryo-EM maps of MuV L_integral_–P and L_body_–P to improve the interpretability, after post-processing in DeepEMhancer^69^. These are also utilized for other figure preparation. Models in (**c**, **e**) are the composite atomic models of MuV L_integral_–P and L_body_–P via rigid body docking of individual models into their respective composite cryo-EM maps, which are B-factor sharpened.

In MuV L_integral_–P, the appendage is also less resolved compared with the body. To improve the resolution, “annealing,” as a facile and robust approach to synchronize proteins^55^, was applied to the same batch of purified MuV L–P samples before the grid preparation. Intriguingly, the particle proportion of L_integral_–P increases from 30.9% to 37.5% after annealing (Supplementary Fig. 2). We then combined L_integral_–P particles from both unannealed and annealed particles and finally obtained a 3.02 Å cryo-EM structure (Table 1 and Supplementary Fig. 3a–d). All five domains of L and regions of P_OD_, P_Linker2_, and P_CTD_ involved in L–P interfaces were clearly resolved (Fig. 1a–c and Supplementary Fig. 4a–c). Following the same strategy, L_body_–P was determined at the resolution of 3.01 Å, and only RdRp and PRNTase are visible in L (Fig. 1d,e, Table 1, and Supplementary Fig. 3e,f). The overall architecture of RdRp and PRNTase in L_body_–P is very similar to the counterparts in MuV L_integral_–P, PIV-5, and VSIV^15,23^.

**Table 1:** Cryo-EM data collection, refinement, and validation statistics of MuV L_integral_–P and L_body_–P

	L_integral_–P	RdRp-PRNTase of L_integral_–P	CD-MTase-CTD of L_integral_–P	P of L_integral_–P	L_body_–P	P of L_body_–P
**Data collection and processing**						
Voltage (kV)	300	300	300	300	300	300
Electron exposure (e^–^/Å^2^)	50	50	50	50	50	50
Defocus range (μm)	–1.5 to –2.5	–1.5 to –2.5	–1.5 to –2.5	–1.5 to –2.5	–1.5 to –2.5	–1.5 to –2.5
Symmetry imposed	C1	C1	C1	C1	C1	C1
Initial particle images(no.)	2,087,570	2,087,570	2,087,570	2,087,570	2,087,570	2,087,570
Final particle images (no.)	438,014	438,014	438,014	88,107	477,568	41,168
Pixel size (Å)	0.53	0.53	0.53	1.06	0.53	1.06
Map resolution (Å)	3.02	2.93	3.13	3.49	3.01	3.63
FSC threshold	0.143	0.143	0.143	0.143	0.143	0.143
Map sharpening B factor (Å^2^)	136.8	133.6	156.1	132.3	143.6	123.1
EMDB code	37957	37959	37958	37960	37961	37962
		Composite map EMD-35864			Composite map EMD-37964	
**Model building and refinement**						
Initial model used (PDB code)	/	6V85	/	4EIJ	6V85	4EIJ
Model composition						
Non-hydrogen atoms	/	11,632	5,588	2,096	10,784	1,958
Protein residues	/	1,464	699	278	1,350	258
Ligands	/	2	0	0	2	0
B factors (Å^2^)						
Protein	/	61.76	76.74	115.85	58.08	130.10
Ligand	/	147.46	/	/	142.51	/
R.m.s deviations						
Bond lengths (Å)	/	0.005	0.005	0.008	0.004	0.007
Bond angles (°)	/	0.757	0.777	1.234	0.605	1.108
Validation						
MolProbity score	/	1.73	1.66	1.91	1.42	1.80
Clashscore	/	10.29	14.27	17.23	7.74	16.46
Poor rotamer (%)	/	1.22	0.64	1.67	0.33	0.45
Ramachandran plot						
Favored (%)	/	97.31	98.09	98.52	98.21	97.60
Allowed (%)	/	2.69	1.91	1.48	1.79	2.40
Disallowed (%)	/	0.00	0.00	0.00	0.00	0.00
PDB code	/	8YXM	8YXL	8YXO	8YXP	8YXR
		Composite model PDB ID 8IZL			Composite model PDB ID 8X01

**Fig. 2:**
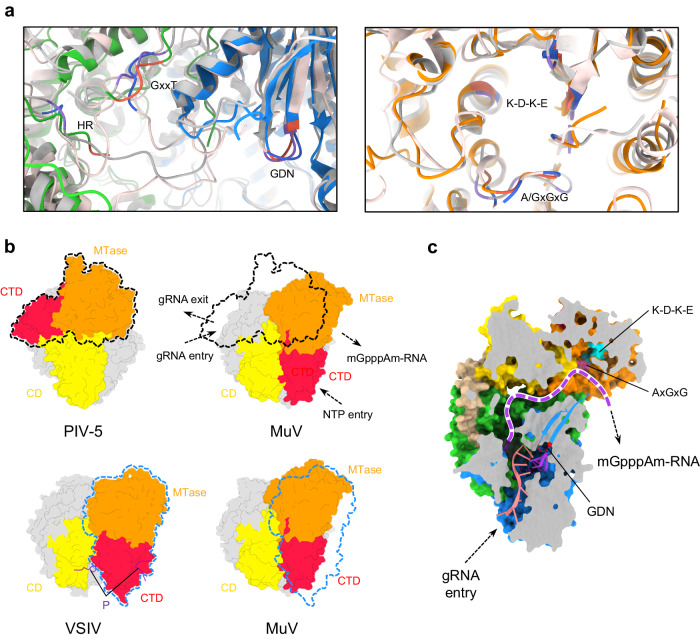
MuV L_integral_–P as a favorable transcription state. **a** Comparison of critical motifs among MuV, PIV-5, and VSIV L. Motifs (GDN, GxxT, HR, K-D-K-E, and A/GxGxG) of MuV, PIV-5, and VSIV are colored in tomato, medium purple, and royal blue, respectively; the other parts of PIV-5 and VSIV are colored in silver and misty rose, respectively. **b** Comparison of CD-MTase-CTD spatial organizations among MuV, PIV-5, and VSIV L. RdRp and PRNTase of all three structures are aligned and colored in light gray. The P fragment of VSIV is colored in purple. The outlines of PIV-5 and VSIV MTase-CTD are depicted in black and blue dashed lines around MuV maps, respectively. **c** Continuous RNA tunnel of MuV L_integral_–P. Superposed nucleotides are from the crystal structure of the reovirus λ3 polymerase initiation complex (PDB ID 1N1H). The purple dashed curve represents the potential elongation path for the transcribed mRNA.

Critical for RNA synthesis, many motifs, including GDN (L_778–780_) motif within RdRp and histidine-arginine (HR, L_1298–1299_) motif within PRNTase, are highly conserved in structures among MuV, PIV-5, and VSIV L–P complexes (Fig. 2a). Two flexible loops termed the priming loop and the intrusion loop in MuV PRNTase have the similar orientations with those of PIV-5^23^. Specifically, the intrusion loop projects into the RNA cavity and the priming loop is oriented to the inner wall of MuV PRNTase (Supplementary Fig. 5a). The up-and-down flipping of these two loops is essential for initiating of RNA synthesis^56^.

### L_integral_–P as a favorable transcription state

Different from the conserved RdRp-PRNTase, CD-MTase-CTD of MuV L_integral_–P adopts a spatial organization distinct from those of PIV-5, though their individual structures are pretty similar (Fig. 2a, b). The detailed alignment showed that MTase and CTD in MuV L_integral_–P appears as an integral on the PRNTase side instead of the RdRp side compared with PIV-5 L. The overall spatial organization of CD-MTase-CTD in MuV L_integral_–P is surprisingly similar to VSIV L (Fig. 2b). VSIV CD-MTase-CTD is highly flexible unless unphosphorylated P locks their configuration^27,57^, while in MuV, no P fragment is resolved around the appendage (Fig. 2b). In MuV L_integral_–P, an α-helix hinge termed Hinge-1 (L_1416–1431_) connecting PRNTase and CD has been visualized (Fig. 1c). It is supposed to provide flexibility to CD positioning via loops flanking it.

Compared with PIV-5, the spatial organization of MuV CD-MTase-CTD renders the helices α53 (L_1439–1458_) and α57 (L_1535–1544_) of CD rotating upward and leaves more space for RNA to access MTase (Supplementary Fig. 6a). Actually, MuV L_integral_–P forms a continuous positively-charged tunnel from the GDN to the K-D-K-E motifs, ideal for the RNA synthesis followed by 5’ capping and methylation, which is favorable as the transcription state (Fig. 2c and Supplementary Fig. 6b). While in PIV-5 L, the RNA tunnel towards the K-D-K-E motif is blocked at the site surrounded by RdRp and CD due to the different spatial organization of CD-MTase-CTD (Supplementary Fig. 6c). Furthermore, the K-D-K-E motif of PIV-5 locates at the outside of the RNA cavity. The capped mRNA is hard and even impossible to get access to the methylation site as the transcription state. MuV L_body_–P owns a flexible appendage, and its RNA tunnel is inaccessible to MTase-CTD, which is unfavorable for transcription. However, the RNA cavity formed by RdRp and PRNTase domains is available, with the potential capability for genome replication (Supplementary Fig. 6d).

### Parallel P tetramers in MuV L–P complex

**Fig. 3:**
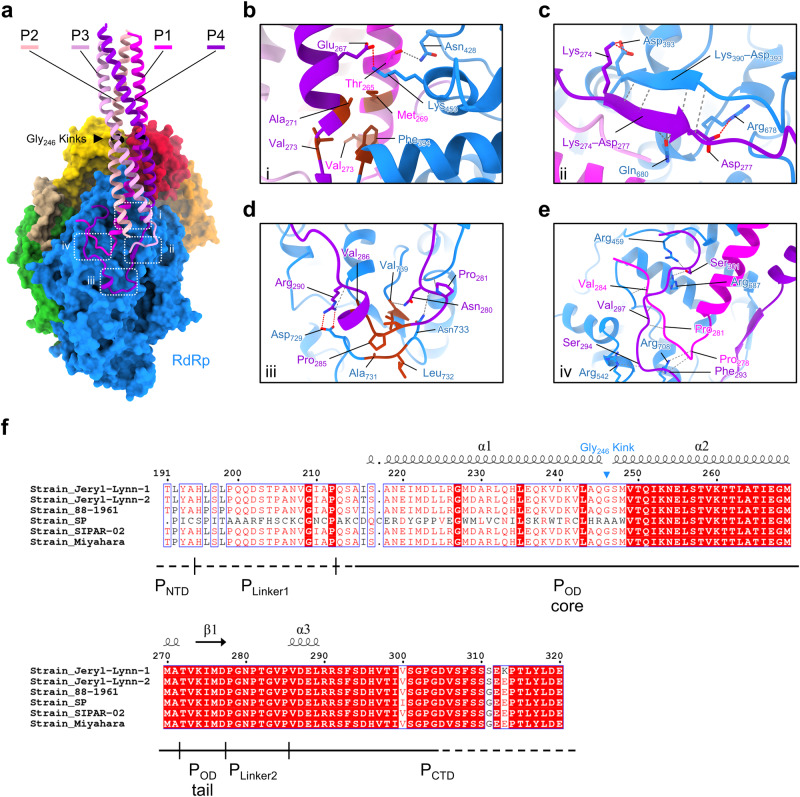
The interface between L an P of MuV polymerase complex. **a** Overall view of tetrameric P bound to RdRp of L. P1, P2, P3, and P4 are colored in magenta, light pink, plum, and dark violet, respectively. Four core interaction zones are boxed with white dotted rectangles. **b–e**, Close-up views of the interaction zones i, ii, iii, and iv. Hydrogen bonds and salt bridges are indicated by dim gray and red dashed lines, respectively. Residues involved in hydrophobic interactions are colored in brown. **f** Sequence alignment of P from six MuV strains spanning residues 191–320. Solid lines beneath the sequences represent the structurally resolved regions. Dashed lines represent the unsolved regions. Labels of secondary structures above the sequences are based on the atomic model of P4. Gly_246_, the kink between helices α1 and α2 of P_OD_, is labeled.

Our L_integral_–P structure revealed four P molecules assembled into a helical bundle around RdRp of L via their respective P_OD_. Different from the previous analysis on MuV P alone^36,41^, four P molecules are more likely to adopt a parallel orientation in L_integral_–P (Fig. 3a and Supplementary Fig. 4b,c). This observation is highly consistent with P molecules in many other nsNSVs^21–24,26,34,58–60^, which indicates a generally conserved mechanism for P molecules to mediate RNA genome replication and transcription.

In cryo-EM maps, four P molecules assemble like a kettle spout stably anchored to L. Our cryo-EM structures capture the clear interface between two P molecules (depicted as P1 and P4, respectively) and L, involving the RdRp, P_OD_, P_Linker2_, and P_CTD_ regions (Fig. 3a). Specifically, P1-Met_269_, P1-Val_273_, P4-Ala_271_, and P4-Val_273_ from the C-terminus of the P_OD_ core region, form a hydrophobic cap to trap the conserved residue L-Phe_394_ (Fig. 3b). A salt bridge between P4-Glu_267_ and L-Lys_453_, together with a hydrogen bond between P1-Thr_265_ and L-Asn_428_, further stabilizes this interface (Fig. 3b). The P_OD_ tail of P4 folds into a β-strand, forming the anti-parallel β-sheet with Lys_390_–Asp_393_ of L (Fig. 3c). Three electrostatic interactions fix both ends of the β-sheet. Furthermore, L-Gln_680_ forms hydrogen bonds with P4-Met_276_ and P4-Asp_277_, enabling close contact between these two β-strands (Fig. 3c).

Intriguingly, the C-terminal domains of P1 and P4 turn to the template entry side (Fig. 3a). The turning point occurs at the P_Linker2_ region of P4 and is trapped in the hydrophobic groove contributed by L-Ala_731_, L-Leu_732_, and L-Val_739_. Hydrogen bonds and salt bridges stabilize residues flanking the turning point (Fig. 3d). Several hydrogen bonds make an interacting network among the P_Linker2_ of P1, P_CTD_ of P4, and RdRp of L (Fig. 3e). The P_CTD_ of P4 strides over P1, forming hydrogen bonds among P4-Ser_301_, L-Arg_459_, and L-Arg_687_. The P_Linker2_ of P1 is surrounded by both L and P4. P1-Thr_282_ and P1-Val_284_ contact with RdRp, and P1-Pro_281_ and P1-Gly_283_ interact with the CTD of P4 (Fig. 3e).

Apparently, L–P binding involves abundant residues via forming a complicated and stable interface^39,61^. Residues from 249 to 299 of P are the major region interacting with L (Supplementary Fig. 7). Interestingly, these fragments in six different MuV strains are identical while other regions are not (Fig. 3f and Supplementary Fig. 8). This indicates that these residues are evolutionally highly conserved, and play critical roles in the stable assembly of the L–P complex.

### Diverse origins of L-binding P_XD_

RNA synthesis requires the advance of L on NC, both of which are tethered by different P_XD_ in the P tetramer^39^. In PIV-5, one P_XD_ binds to RdRp as the major contact site, preventing the detachment of P from the L–P complex^23^. There are four P molecules in PIV-5 L–P, and the exact origin of this L-binding P_XD_ remains vague due to the poor densities of P_OD_ tail, P_Linker2_, and P_CTD_. In reference to the HRSV L–P structure, the authors speculated that this L-binding P_XD_ in PIV-5 belongs to P4 as well^21,23^.

**Fig. 4:**
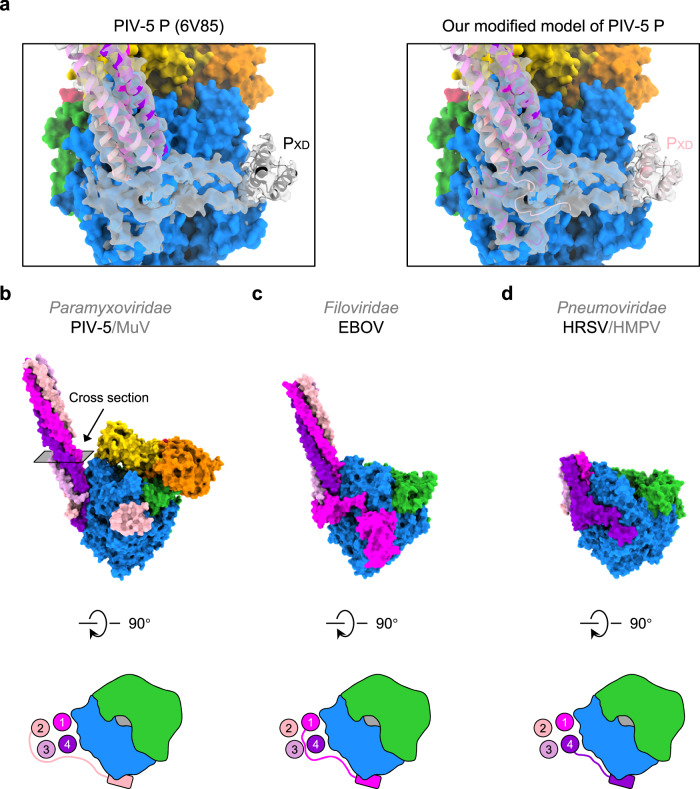
Diverse origins of L-binding P_XD_ in nsNSVs. **a** The atomic model of PIV-5 P (PDB ID 6V85) docked into the PIV-5 P density (EMD-21095) (Left) and the modified atomic model of PIV-5 P based on the atomic model of MuV P (Right). **b** The side view of our newly built atomic model of PIV-5 L–P complex. The cartoon demonstrates the top view of the L–P interface. Four circles represent the cross-sections of the P tetramer. The rounded rectangle represents the P_XD_. **c** The side view of the atomic model of the EBOV L–P complex. The top view of the L–P interface is shown in the cartoon style. **d** The side view of the atomic model of the HRSV L–P complex. The top view of the L–P interface is shown in the cartoon style.

PIV-5 has high sequence identity (L: 58.7%; P: 37.0%) and structural similarity with MuV (Supplementary Fig. 9). Thus, we docked the atomic model of MuV P into the EM density of PIV-5 P (EMD-21095). Interestingly, MuV P1 and P4 fit well in the density map of PIV-5 (Supplementary Fig. 4d). Our intensive model building on PIV-5 identifies that C-terminal domains of P1 and P4 orient to the template entry side instead of the NTP entry side (Fig. 4a). In PIV-5, the L-binding P_XD_ on the NTP entry side should not belong to P4, but P2 (Fig. 4a, b). An extensive survey on the origin of P_XD_ among nsNSVs shows that the L-binding P_XD_ of EBOV belongs to P1 (Fig. 4c), while L-binding P_XD_-like regions of HRSV and HMPV belong to P4^21,22,24,26^ (Fig. 4d). Apparently, L-binding P_XD_ has diverse origins.

**Fig. 5:**
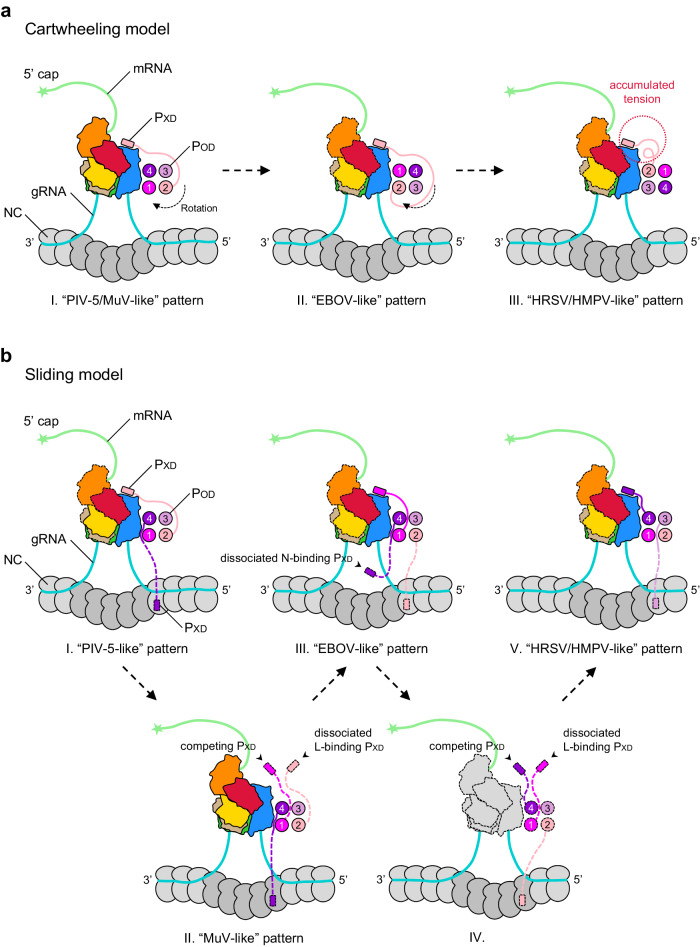
Models for RNA synthesis of nsNSVs. **a** The revised cartwheeling model. Tetrameric P_OD_ self-rotates during the advance of L along the RNA template, with the same P_XD_ attached to RdRp. Meanwhile, other P_XD_ may dynamically bind to N to cartwheel on NC. After several rounds, the coiling tension of the P_CTD_ region will arise. The L–P complexes shown here are observed from the top view of the architecture in Fig. 1b. Domains that have not yet been determined are outlined in dashed lines. **b** The sliding model. One P_XD_ binds to RdRp while the other P_XD_ attaches to NC. During the advance of L, the L-binding P_XD_ may be released, and then one of the four P_XD_ competes to rebind L. Once the new L-P_XD_ interaction forms, the remaining P_XD_ may bind N in proximity, followed by cycle repeats.

The traditional cartwheeling model assumes that the relative position of L-binding P_XD_ and its corresponding P_OD_ stays the same during rotation cycles^38,47–50^. The origin diversity of P_XD_ mentioned above seemingly supports an optimized cartwheeling mechanism, by which the rotation of P_OD_ does not interfere with the stable binding of one single P_XD_ to RdRp (Fig. 5a). Nevertheless, after several rotation cycles, the coiling tension will accumulate in L-binding P_XD_, which may break the stable interface formed by P_XD_ and RdRp and further hamper the binding sustainability.

Different from the cartwheeling model, another popular sliding model claims that any P_XD_ in tetrameric P can stably bind to RdRp, and other P_XD_ will reengage with RdRp only after the falling-off of the current P_XD_ from L^39^. Diverse origins of P_XD_ in nsNSVs are well consistent with the proposed free competition among all four L binding-competent P_XD_ (Fig. 5b)_._ The other P_XD_ dynamically binds to nucleoproteins, ensuring the processivity of the RNA synthesis.

## Discussion

L–P complex is responsible for RNA synthesis in both replication and transcription processes in nsNSVs. As the core component of the L–P complex, one L structure is usually resolved from each viral species. However, L differs in structure among different species, especially on the spatial organization of CD-MTase-CTD. Via cryo-EM, we resolved two conformations of MuV L–P complex: L_body_–P and L_integral_–P. MuV L_integral_–P adopts a different spatial organization of CD, MTase, and CTD from PIV-5 L–P and possesses a continuous RNA tunnel as the transcriptionally competent form. The proximity of MTase during the elongation of the transcription ensures the regulation of both the methylation and polyadenylation process^20,62^.

Once the polymerase adopts the replication state, the K-D-K-E motif and SAM-binding motif in MTase of L are no longer required. MuV L_integral_–P may bypass the MTase domain as one possible replication form (Supplementary Fig. 10a). MuV L_body_–P takes an appendage-free conformation but still owns an exposed RNA cavity formed by RdRp and PRNTase domains with the potential to another replication state for RNA synthesis (Supplementary Fig. 6d and 10b). In PIV-5, the RNA tunnel to MTase is blocked, while RNA can still come out from the pore formed by RdRp and CD. Thus, the conformation revealed in PIV-5 might be the third form as the replication state (Supplementary Fig. 10c). Validation of different forms for replication or transcription needs the further setup of functional assays on various mutants.

In both replication and transcription processes, the priming loop and the intrusion loop are critical to regulating RNA synthesis^56^. Both MuV and PIV-5 belong to the family *Paramyxoviridae* and have the exact position in the priming loop and intrusion loop. Other species within the same family also share the conserved positions of these two loops (Supplementary Fig. 11). To date, HRSV and HMPV in *Pneumoviridae* harbor both up-flipping loops^21,24^ (Supplementary Fig. 5b). The priming loops of VSIV and RABV in the *Rhabdoviridae* occupy the cavity, while the intrusion loops closely attach to PRNTase^15,25^ (Supplementary Fig. 5c). We hypothesize that the genetic diversity among families results in textural differences in cavities, further leading to these three preferred organizations. Previous studies proposed the possible connection between the positions of two loops and the polymerase states of RNA synthesis. Based on the EBOV P–L–RNA structure, during the elongation state, the priming loop and the intrusion loop retract from the cavity to accommodate RNA^26^ (Supplementary Fig. 5d). For the resting state, two loops of apo-L either adopt those three stabilized patterns or wobble in the empty cavity, waiting for the incoming NTP and RNA to transform into the pre-initiation state resembling L structures of VSIV and RABV^25,27^.

P is required for RNA synthesis in most nsNSVs. MuV P forms parallel dimers and further self-assembles into anti-parallel tetramers in the case of the recombinant P_OD_^36,41^. In this study, we observed that P tetramerizes more probably in a parallel pattern when constituted in complex with L. Due to the moderate resolution of P tetramer, we could not recognize the kink at Gly_246_, the unique feature to identify the helix orientation, and we could not rule out the possibility of MuV P tetramer in an anti-parallel configuration. The oligomerization forms of P in nsNSVs may depend on different conditions. Nipah virus P_OD_ assembles into trimers in solution but is crystallized into tetramers^34,60,63^. Crystal structures of the Zaire ebolavirus VP35 oligomerization domain are trimers, whereas VP35 forms tetramers in polymerase complexes^26,64^. Therefore, the involvement of L may guide the assembly of P monomers in EBOV, MuV, and others.

Based on parallel P tetramers in nsNSVs L–P complexes, the cartwheeling or sliding model has been proposed to describe the advance of polymerase on NC^39,46–49^. In this study, diverse origins of P_XD_ in nsNSVs provide direct clues to the rotation of P molecules, though from different species. Compared with the cartwheeling model, the sliding model seems more plausible based on current biochemical and structural evidence. The comprehensive structural analyses on the L–P–N–RNA super-complex with modified L or P will be helpful in verifying either the cartwheeling or sliding model. Further studies will shed light on anti-viral drug discovery and eventually benefit human health.

## Methods

### Protein expression and purification

The mumps virus (strain Jeryl-Lynn) L gene (Genbank: AAF70396.1) with an N-terminal dual Strep-tag II and/or MuV P gene (Genbank: AAF70389.1) with a C-terminal Flag-tag were subcloned into the pFastBac Dual vector and expressed in Sf9 cells (Invitrogen, USA). Cells expressing L (for de novo RNA synthesis assay) or the L–P complex were lysed by Dounce homogenization in Lysis Buffer (300 mM NaCl, 50 mM Tris-HCl, 6 mM MgCl_2_, and 1 mM TCEP, pH 8.0) supplemented with EDTA-free protease inhibitor cocktail (Bimake, USA). After the high-speed centrifugation at 100,000 *×* *g* for 40 min, the supernatant was incubated with Strep-Tactin (Cytiva, USA) resins for 30 min at 4 °C. The resins were washed using Lysis Buffer and eluted using Elution Buffer (2.5 mM d-Desthiobiotin, 150 mM NaCl, 20 mM Tris-HCl, 6 mM MgCl_2_, and 1 mM TCEP, pH 8.0). The eluted L–P complex was further purified using the captoQ ImpRes column (Cytiva, USA). The fractions containing the L–P complex were concentrated and loaded onto the Superose 6 Increase column (Cytiva, USA) equilibrated in SEC Buffer (150 mM NaCl, 20 mM Tris-HCl, 6 mM MgCl_2_, and 1 mM TCEP, pH 8.0). L and P in the purified complex were verified by the western blotting using the Strep-Tactin horse radish peroxidase conjugate at the dilution of 1:100,000 (IBA Lifesciences, Germany, catalog number 2-1502-001) and mouse monoclonal antibody against the Flag tag at the dilution of 1:1,000 (Sigma, USA, catalog number F1804), respectively. Samples were concentrated, flash-frozen, and stored at –80 °C.

### De novo RNA synthesis assay

De novo RNA synthesis assays were carried out using 200 nM MuV L or L–P complex and 200 nM templates (Le18, 5’-AUUCAUUCUCCCCUUGGU-3’; Tr18, 5’-ACCAAGGGGAGAAAGUAA-3’) as a reaction mixture in the buffer containing 20 mM Tris-HCl, 100 mM NaCl, 6 mM MgCl_2_, and 1 mM TCEP, pH 8.0. The reaction mixtures were incubated for 10 min at room temperature. Reactions were initiated through the addition of an NTP mix (final concentrations, 100 μM each of ATP, UTP, and CTP, 1 μM GTP) and 4 μCi [α-^32^P] GTP (3,000 Ci/mmol; Perkin Elmer, USA), allowed to proceed for 3 h at 30 °C, and then stopped by the addition of 5 μL Stop Buffer (8 M urea, 20 mM EDTA, 0.025% xylene cyanol, and 0.025% bromophenol blue). The samples were boiled for 5 min and immediately cooled on ice for another 5 min, followed by running on a 23% (19:1 acrylamide/bisacrylamide) urea polyacrylamide slab gels in 90 mM Tris-borate (pH 8.0) and 0.2 mM EDTA. The radiograph was obtained by storage-phosphor scanning (Typhoon; Cytiva, USA).

### Primer-extension assay

Primer-extension assays were carried out using 200 nM MuV L–P complex and 200 nM template (Le18, 5’-AUUCAUUCUCCCCUUGGU-3’) in a reaction mixture containing 20 mM Tris-HCl, 100 mM NaCl, 6 mM MgCl_2_, and 1 mM TCEP, pH 8.0. The reaction mixtures were incubated for 10 min at room temperature and then supplemented with the primer (5’-pACCA-3’; final concentration, 1 μM) followed by incubation for 10 min at room temperature. Reactions were initiated by adding 4 μCi [α-^32^P] GTP (3,000 Ci/mmol; Perkin Elmer, USA) and one of the NTP sets: GTP (final concentration, 1 μM GTP), ATP + GTP (final concentrations, 100 μM ATP and 1 μM GTP), UTP + ATP + GTP (final concentrations, 100 μM each of ATP and UTP, and 1 μM GTP). Reactions were allowed to proceed for 3 h at 30 °C and then stopped by adding 5 μL Stop Buffer. The samples were boiled for 5 min and immediately cooled on ice for another 5 min, followed by running on a 23% (19:1 acrylamide/bisacrylamide) urea polyacrylamide slab gels in 90 mM Tris-borate (pH 8.0) and 0.2 mM EDTA. The radiograph was obtained by storage-phosphor scanning (Typhoon; Cytiva, USA).

### Cryo-EM sample preparation

MuV L–P complex at 2.0 μM was melted on ice. Part of the sample was kept as the untreated sample under 4 °C for vitrification. The other part of the sample was subjected to the annealing treatment as described^55^. Specifically, no more than 20 μL of the aliquot was pipetted into one PCR tube and heated in the 37 °C water bath for 1 min. The heated sample was immediately immersed in a mixture of salt, ice, and water (measured temperature: −18 °C) for 20 s and then transferred into the ice bath (measured temperature: 0 °C) for 2 min.

The annealed and unannealed samples were applied to glow-discharged holey grids R2/1 (Quantifoil, Ted Pella, USA). The grids were blotted using a Vitrobot Mark IV (Thermo Fisher Scientific, USA) with 1 s blotting time, force level of 0, and humidity of 100% at 4 °C, and then immediately plunged into liquid ethane and transferred to liquid nitrogen for future cryo-EM imaging.

### Cryo-EM data collection

Data collection was performed with the Titan Krios G^3*i*^ microscope (Thermo Fisher Scientific, USA) equipped with a K3 BioQuantum direct electron detector (Gatan, USA). Movies were collected via FEI EPU (Thermo Fisher Scientific, USA) automated data collection software at a total dose of ~50 e^−^/Å^2^ fractionated over 40 frames with defocus values ranging from −1.5 to −2.5 μm. A super-resolution mode was used with the final pixel size at 0.53 Å.

### Cryo-EM data processing

The raw movie stacks of both the annealed group and the unannealed group were aligned and summed in accordance with dose weighting with MotionCor2.1^65^. The contrast transfer function (CTF) parameters of the summed micrographs were determined with CTFFIND4^66^. Micrographs of two groups with maximum resolution estimates better than 5 Å were imported into CryoSPARC v3.1, respectively^67^. Automatic particle picking was performed on the selected micrographs, and particle sets were created and subjected to reference-free 2D classifications. Obvious junks were excluded from the particle set. After rounds of 2D classifications, 1,172,627 particles (the annealed group) and 914,943 particles (the unannealed group) were selected for the *Ab*-*Initio* 3D reconstruction, respectively.

Two of three classes for each group were selected as reference structures for the following heterogeneous refinement. In both groups, one class (termed L_integral_–P) contains more structural information about L, while the other class (termed L_body_–P) contains less. The particle proportion of L_integral_–P in the annealed group is 37.5%, while in the unannealed group is 30.9%. The class L_integral_–P in both groups shows no noticeable difference in structure. Therefore, we combined the L_integral_–P or L_body_–P datasets from both groups to improve the resolution. After B-factor sharpening, the respective resolutions of L_integral_–P and L_body_–P were estimated at 3.02 Å and 3.01 Å, based on the gold-standard Fourier shell correlation (FSC) 0.143.

To improve the resolution of L_integral_–P, the local refinements were applied on the body and the appendage via generating focused maps. P tetramers in both L_integral_–P and L_body_–P were poorly resolved; particles were re-extracted using P as the box centers and then subjected to 3D refinements. More cryo-EM densities of P were visible in L_integral_–P and L_body_–P. The locally refined maps, including the body, the appendage, and P tetramers of L_integral_–P, were B-factor sharpened at their respective resolutions of 2.93 Å, 3.13 Å, and 3.49 Å. The locally refined map of P tetramers of L_body_–P was estimated at the resolution of 3.63 Å.

For both L_integral_–P and L_body_–P, we combined their respective globally and locally refined maps, including the body, P tetramers, and/or the appendage, into the composite maps using the phenix.combine_focused_map in PHENIX 1.20.1^68^. These composite maps were B-factor sharpened for the rigid body docking of individual atomic models or post-processed using the DeepEMhancer to improve their interpretability for figure preparation^69^. The DeepEMhancer processed maps, together with their locally refined maps, were deposited in the EMDB.

### Model building and structural analysis

The homology model of MuV RdRp and PRNTase of L was generated using PIV-5 L (PDB ID 6V85) as the reference in SWISS-MODEL^70^, and the model of CD-MTase-CTD was predicted by RoseTTAFold^71^. These two models were separately docked as rigid bodies into the locally refined maps of L_integral_–P and L_body_–P using UCSF ChimeraX 1.5^72^, manually adjusted in COOT 0.9.7^73^, and real-space refined against their respective locally refined maps in PHENIX 1.20.1^68^. The stereochemical quality of each model was assessed using MolProbity^74^.

The crystal structure of MuV (strain 88-1961) P_OD_ (PDB ID 4EIJ) was used to guide the manual building of MuV P. The rigid body docking of the anti-parallel tetrameric P_OD_ crystal structure was attempted. P_OD_ core fragments (P_249–271_) of P1 and P4 were successfully docked, but the helices of P2 and P3 failed to fit accurately into the density. We then inverted the orientation of the P2–P3 dimer, yielding a reasonable coordinate of the parallel P tetramer. The final coordinates were real-space refined against their respective locally refined maps in PHENIX 1.20.1^68^. The stereochemical quality of each model was assessed using MolProbity^74^. The atomic models of P tetramer, together with the body and/or the appendage, were docked as rigid bodies into their respective composite maps of L_integral_–P and L_body_–P with the assistance of their globally refined maps; the composite atomic models for L_integral_–P and L_body_–P were built.

The ambiguous P density of PIV-5 was also built based on our homologous structure of MuV P. The PIV-5 P_OD_ tetramer was shifted towards its N-terminus direction for one turn of the α-helix. Four fragments of varying lengths were extended from the end of the P_OD_ core (P_201–272_) to fit the previously unmodeled density.

Structural analyses, including surface electrostatic distribution and structural superimposition, were fulfilled in UCSF ChimeraX. The L–P interface was analyzed using the PDBePISA 1.48^75^.

Protein sequences were aligned by Clustal Omega^76^ and presented using ESPript 3.0^77^. The phylogenetic tree was generated via the neighbor-joining method with bootstrap values determined by 1000 replicates in MEGA 11^78^.

### Reporting summary

Further information on research design is available in the Nature Portfolio Reporting Summary linked to this article.

### Supplementary information

Supplementary Information

Peer Review File

Reporting Summary

### Source data

Source Data

## Data Availability

The cryo-EM density maps, including the globally refined maps, locally refined maps, and the DeepEMhancer processed composite maps, have been deposited to the Electron Microscopy Data Bank (EMDB, https://www.ebi.ac.uk/pdbe/emdb/). The atomic coordinates corresponding to the locally refined maps and the composite maps of MuV L_integral_–P and L_body_–P have been deposited to the Protein Data Bank (PDB, https://www.rcsb.org/). The accession numbers are listed as follows: EMD-37957 (L_integral_–P as the whole), EMD-37959 and 8YXM (RdRp-PRNTase of L_integral_–P), EMD-37958 and 8YXL (CD-MTase-CTD of L_integral_–P), EMD-37960 and 8YXO (P of L_integral_–P), and EMD-35864 and 8IZL (the composite map of L_integral_–P from EMD-37959, EMD-37958, and EMD-37960 and the composite model from PDB IDs 8YXM, 8YXL and 8YXO); EMD-37961 and 8YXP (L_body_–P as the whole), EMD-37962 and 8YXR (P of L_body_–P), and EMD-37964 and 8X01 (the composite map of L_body_–P from EMD-37961 and EMD-37962 and the composite model from PDB IDs 8YXP and 8YXR). Details are listed in Table 1 and Supplementary Fig. 2. All other data is available in the main manuscript file and/or the supplementary information. Source data are provided with this paper.

## References

[1] Knipe, D. & Howley, P. Fields virology 5th edition. (Philadelphia: Lippincott Williams & Wilkins, 2007).

[2] Kuhn JH (2010). Proposal for a revised taxonomy of the family Filoviridae: classification, names of taxa and viruses, and virus abbreviations. Arch. Virol..

[3] Patton JT, Davis NL, Wertz GW (1984). N protein alone satisfies the requirement for protein synthesis during RNA replication of vesicular stomatitis virus. J. Virol..

[4] Arnheiter H, Davis NL, Wertz G, Schubert M, Lazzarini RA (1985). Role of the nucleocapsid protein in regulating vesicular stomatitis virus RNA synthesis. Cell.

[5] Song X (2019). Self-capping of nucleoprotein filaments protects the Newcastle disease virus genome. Elife.

[6] Follett EA, Pringle CR, Wunner WH, Skehel JJ (1974). Virus replication in enucleate cells: vesicular stomatitis virus and influenza virus. J. Virol..

[7] Grosfeld H, Hill MG, Collins PL (1995). RNA replication by respiratory syncytial virus (RSV) is directed by the N, P, and L proteins; transcription also occurs under these conditions but requires RSV superinfection for efficient synthesis of full-length mRNA. J. Virol..

[8] Emerson SU, Wagn er,RR (1973). L protein requirement for in vitro RNA synthesis by vesicular stomatitis virus. J. Virol..

[9] Hercyk N, Horikami SM, Moyer SA (1988). The vesicular stomatitis virus L protein possesses the mRNA methyltransferase activities. Virology.

[10] Feldmann H, Sprecher A, Geisbert TW (2020). Ebola. N. Engl. J. Med..

[11] Ogino T, Kobayashi M, Iwama M, Mizumoto K (2005). Sendai virus RNA-dependent RNA polymerase L protein catalyzes cap methylation of virus-specific mRNA. J. Biol. Chem..

[12] Emerson SU, Yu Y, Both NS (1975). and L proteins are required for in vitro RNA synthesis by vesicular stomatitis virus. J. Virol..

[13] Poch O, Sauvaget I, Delarue M, Tordo N (1989). Identification of four conserved motifs among the RNA-dependent polymerase encoding elements. EMBO J..

[14] Poch O, Blumberg BM, Bougueleret L, Tordo N (1990). Sequence comparison of five polymerases (L proteins) of unsegmented negative-strand RNA viruses: theoretical assignment of functional domains. J. Gen. Virol..

[15] Liang B (2015). Structure of the L Protein of Vesicular Stomatitis Virus from Electron Cryomicroscopy. Cell.

[16] Ogino T, Banerjee AK (2007). Unconventional mechanism of mRNA capping by the RNA-dependent RNA polymerase of vesicular stomatitis virus. Mol. Cell.

[17] Ogino T, Banerjee AK (2008). Formation of guanosine(5’)tetraphospho(5’)adenosine cap structure by an unconventional mRNA capping enzyme of vesicular stomatitis virus. J. Virol..

[18] Grdzelishvili VZ (2005). A single amino acid change in the L-polymerase protein of vesicular stomatitis virus completely abolishes viral mRNA cap methylation. J. Virol..

[19] Li J, Fontaine-Rodriguez EC, Whelan SP (2005). Amino acid residues within conserved domain VI of the vesicular stomatitis virus large polymerase protein essential for mRNA cap methyltransferase activity. J. Virol..

[20] Li J, Rahmeh A, Brusic V, Whelan SP (2009). Opposing effects of inhibiting cap addition and cap methylation on polyadenylation during vesicular stomatitis virus mRNA synthesis. J. Virol..

[21] Gilman MSA (2019). Structure of the Respiratory Syncytial Virus Polymerase Complex. Cell.

[22] Cao D (2020). Cryo-EM structure of the respiratory syncytial virus RNA polymerase. Nat. Commun..

[23] Abdella R, Aggarwal M, Okura T, Lamb RA, He Y (2020). Structure of a paramyxovirus polymerase complex reveals a unique methyltransferase-CTD conformation. Proc. Natl Acad. Sci..

[24] Pan J (2019). Structure of the human metapneumovirus polymerase phosphoprotein complex. Nature.

[25] Horwitz JA, Jenni S, Harrison SC, Whelan SPJ (2020). Structure of a rabies virus polymerase complex from electron cryo-microscopy. Proc. Natl Acad. Sci..

[26] Yuan B (2022). Structure of the Ebola virus polymerase complex. Nature.

[27] Jenni S (2020). Structure of the Vesicular Stomatitis Virus L Protein in Complex with Its Phosphoprotein Cofactor. Cell Rep..

[28] Canter DM, Perrault J (1996). Stabilization of vesicular stomatitis virus L polymerase protein by P protein binding: a small deletion in the C-terminal domain of L abrogates binding. Virology.

[29] Bloyet LM (2016). HSP90 Chaperoning in Addition to Phosphoprotein Required for Folding but Not for Supporting Enzymatic Activities of Measles and Nipah Virus L Polymerases. J. Virol..

[30] Mellon MG, Emerson SU (1978). Rebinding of transcriptase components (L and NS proteins) to the nucleocapsid template of vesicular stomatitis virus. J. Virol..

[31] Morin B, Rahmeh AA, Whelan SP (2012). Mechanism of RNA synthesis initiation by the vesicular stomatitis virus polymerase. EMBO J..

[32] Horikami SM, Curran J, Kolakofsky D, Moyer SA (1992). Complexes of Sendai virus NP-P and P-L proteins are required for defective interfering particle genome replication in vitro. J. Virol..

[33] La Ferla FM, Peluso RW (1989). The 1:1 N-NS protein complex of vesicular stomatitis virus is essential for efficient genome replication. J. Virol..

[34] Bruhn JF (2014). Crystal structure of the nipah virus phosphoprotein tetramerization domain. J. Virol..

[35] Communie G (2013). Structure of the tetramerization domain of measles virus phosphoprotein. J. Virol..

[36] Cox R (2013). Structural and functional characterization of the mumps virus phosphoprotein. J. Virol..

[37] Cox R (2014). Structural studies on the authentic mumps virus nucleocapsid showing uncoiling by the phosphoprotein. Proc. Natl Acad. Sci..

[38] Curran J, Boeck R, Lin-Marq N, Lupas A, Kolakofsky D (1995). Paramyxovirus phosphoproteins form homotrimers as determined by an epitope dilution assay, via predicted coiled coils. Virology.

[39] Du Pont V, Jiang Y, Plemper RK (2019). Bipartite interface of the measles virus phosphoprotein X domain with the large polymerase protein regulates viral polymerase dynamics. PLoS Pathog..

[40] Kingston RL, Baase WA, Gay LS (2004). Characterization of nucleocapsid binding by the measles virus and mumps virus phosphoproteins. J. Virol..

[41] Pickar A (2015). Oligomerization of Mumps Virus Phosphoprotein. J. Virol..

[42] Galloux M (2012). Characterization of a viral phosphoprotein binding site on the surface of the respiratory syncytial nucleoprotein. J. Virol..

[43] Communie G (2013). Atomic resolution description of the interaction between the nucleoprotein and phosphoprotein of Hendra virus. PLoS Pathog..

[44] Longhi S (2003). The C-terminal Domain of the Measles Virus Nucleoprotein Is Intrinsically Disordered and Folds upon Binding to the C-terminal Moiety of the Phosphoprotein. J. Biol. Chem..

[45] Bernard C (2009). Interaction between the C-terminal domains of N and P proteins of measles virus investigated by NMR. FEBS Lett..

[46] Curran J (1998). A role for the Sendai virus P protein trimer in RNA synthesis. J. Virol..

[47] Kolakofsky D (2016). Paramyxovirus RNA synthesis, mRNA editing, and genome hexamer phase: A review. Virology.

[48] Kolakofsky D (2021). Sendai virus and a unified model of mononegavirus RNA synthesis. Viruses.

[49] Kolakofsky D, Le Mercier P, Iseni F, Garcin D (2004). Viral DNA polymerase scanning and the gymnastics of Sendai virus RNA synthesis. Virology.

[50] Krumm SA, Takeda M, Plemper RK (2013). The measles virus nucleocapsid protein tail domain is dispensable for viral polymerase recruitment and activity. J. Biol. Chem..

[51] Hviid A, Rubin S, Mühlemann K (2008). Mumps. Lancet.

[52] Bockelman C, Frawley TC, Long B, Koyfman A (2018). Mumps: An emergency medicine-focused update. J. Emerg. Med..

[53] Mourez T, Dina J (2018). Mumps virus: a comprehensive review. Virologie (Montrouge).

[54] Kingston RL, Gay LS, Baase WS, Matthews BW (2008). Structure of the nucleocapsid-binding domain from the mumps virus polymerase; an example of protein folding induced by crystallization. J. Mol. Biol..

[55] Chu X (2022). Annealing synchronizes the 70S ribosome into a minimum-energy conformation. Proc. Natl Acad. Sci. USA.

[56] Ogino M, Gupta N, Green TJ, Ogino T (2019). A dual-functional priming-capping loop of rhabdoviral RNA polymerases directs terminal de novo initiation and capping intermediate formation. Nucleic Acids Res.

[57] Gould JR (2021). Consequences of Phosphorylation in a Mononegavirales Polymerase-Cofactor System. J. Virol..

[58] Tarbouriech N, Curran J, Ruigrok RW, Burmeister WP (2000). Tetrameric coiled coil domain of Sendai virus phosphoprotein. Nat. Struct. Biol..

[59] Yabukarski F (2014). Structure of Nipah virus unassembled nucleoprotein in complex with its viral chaperone. Nat. Struct. Mol. Biol..

[60] Beltrandi M (2015). Insights into the coiled-coil organization of the Hendra virus phosphoprotein from combined biochemical and SAXS studies. Virology.

[61] Bowman MC, Smallwood S, Moyer SA (1999). Dissection of individual functions of the Sendai virus phosphoprotein in transcription. J. Virol..

[62] Rahmeh AA, Li J, Kranzusch PJ, Whelan SP (2009). Ribose 2’-O methylation of the vesicular stomatitis virus mRNA cap precedes and facilitates subsequent guanine-N-7 methylation by the large polymerase protein. J. Virol..

[63] Bruhn JF, Hotard AL, Spiropoulou CF, Lo MK, Saphire EO (2019). A conserved basic patch and central kink in the nipah virus phosphoprotein multimerization domain are essential for polymerase function. Structure.

[64] Zinzula L (2019). Structures of Ebola and Reston Virus VP35 oligomerization domains and comparative biophysical characterization in all ebolavirus species. Structure.

[65] Zheng SQ (2017). MotionCor2: anisotropic correction of beam-induced motion for improved cryo-electron microscopy. Nat. Methods.

[66] Rohou A, Grigorieff N (2015). CTFFIND4: Fast and accurate defocus estimation from electron micrographs. J. Struct. Biol..

[67] Punjani A, Rubinstein JL, Fleet DJ, Brubaker MA (2017). cryoSPARC: Agorithms for rapid unsupervised cryo-EM structure determination. Nat. Methods.

[68] Afonine PV (2018). Real-space refinement in PHENIX for cryo-EM and crystallography. Acta Crystallogr D. Struct. Biol..

[69] Sanchez-Garcia R (2021). DeepEMhancer: A deep learning solution for cryo-EM volume post-processing. Commun. Biol..

[70] Waterhouse A (2018). SWISS-MODEL: Homology modelling of protein structures and complexes. Nucleic Acids Res..

[71] Baek M (2021). Accurate prediction of protein structures and interactions using a three-track neural network. Science.

[72] Pettersen EF (2021). UCSF ChimeraX: Structure visualization for researchers, educators, and developers. Protein Sci..

[73] Emsley P, Cowtan K (2004). Coot: model-building tools for molecular graphics. Acta Crystallogr D. Biol. Crystallogr.

[74] Chen VB (2010). MolProbity: all-atom structure validation for macromolecular crystallography. Acta Crystallogr D. Biol. Crystallogr.

[75] Krissinel E, Henrick K (2007). Inference of macromolecular assemblies from crystalline state. J. Mol. Biol..

[76] Sievers F (2011). Fast, scalable generation of high-quality protein multiple sequence alignments using Clustal Omega. Mol. Syst. Biol..

[77] Robert X, Gouet P (2014). Deciphering key features in protein structures with the new ENDscript server. Nucleic Acids Res..

[78] Tamura K, Stecher G, Kumar S (2021). MEGA11: Molecular evolutionary genetics analysis version 11. Mol. Biol. Evol..

