# Supplementary material for: Structures of the mumps virus polymerase complex via cryo-electron microscopy
Source: Nat Commun. 2024 May 17;15:4189. doi: 10.1038/s41467-024-48389-9 (PMC11101452; doi:10.1038/s41467-024-48389-9)
Supplement: Supplementary file 1 — Supplementary Information [file 41467_2024_48389_MOESM1_ESM.pdf]

Structures of the mumps virus polymerase complex via cryo-electron microscopy

Tianhao Li[1,2,3,4,8], Mingdong Liu[1,2,3,8], Zhanxi Gu[5,6], Xin Su[1,2,3,7], Yunhui Liu[1,2,3], Jinzhong Lin[7], Yu Zhang[6], Qing-Tao Shen[1,2,3,4*]

Affiliations:

[1]School of Life Sciences, Department of Chemical Biology, Southern University of Science and Technology, Shenzhen 518055, China.

[2]Laboratory for Marine Biology and Biotechnology, Qingdao National Laboratory for Marine Science and Technology, Qingdao 266237, China.

[3]Institute for Biological Electron Microscopy, Southern University of Science and Technology, Shenzhen, 518055, China.

[4]School of Life Science and Technology, ShanghaiTech University, Shanghai 201210, China.

[5]University of Chinese Academy of Sciences, Beijing 100049, China.

[6]Key Laboratory of Synthetic Biology, Center for Excellence in Molecular Plant Sciences, Institute of Plant Physiology and Ecology, Chinese Academy of Sciences, Shanghai 200032, China.

[7]State Key Laboratory of Genetic Engineering, School of Life Sciences, Zhongshan Hospital, Fudan University, Shanghai, 200438, China.

[8]These authors contributed equally: Tianhao Li, Mingdong Liu

[*]Correspondence to shenqt@sustech.edu.cn (Q.-T.S.)

Supplementary Materials:

Supplementary Fig.1 to Fig.11

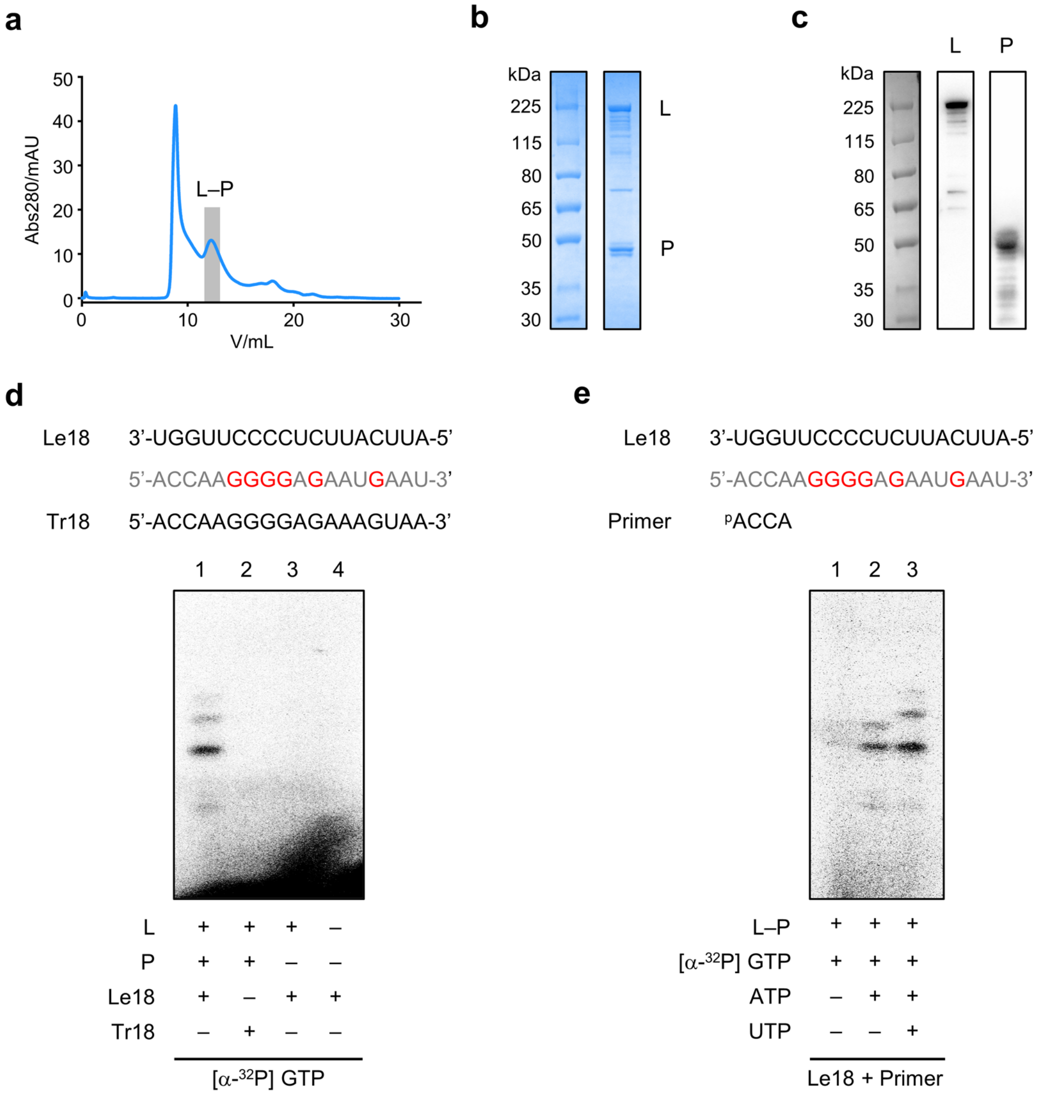

Supplementary Fig. 1 Purification and verification of MuV L–P complex. a, Size-exclusive chromatogram of MuV L–P. The grey rectangle indicates the fractions collected for cryo-EM sample preparation and verifications in **b**–**e**. **b,c,** The SDS-PAGE gel and western blotting profiles of purified MuV L–P complex. **d,** The *de novo* RNA synthesis assay of MuV L–P complex. The first 18 nucleotides of the leader sequence (Le18) are used as a template. The control is the 18 nucleotides at the 5' end of the genome (Tr18). **e,** The primer-extension assay of MuV L–P complex. A 4-nt primer was incubated with the Le18 template before initiating the reaction. Source data in **a**–**e** are provided as a Source Data file.

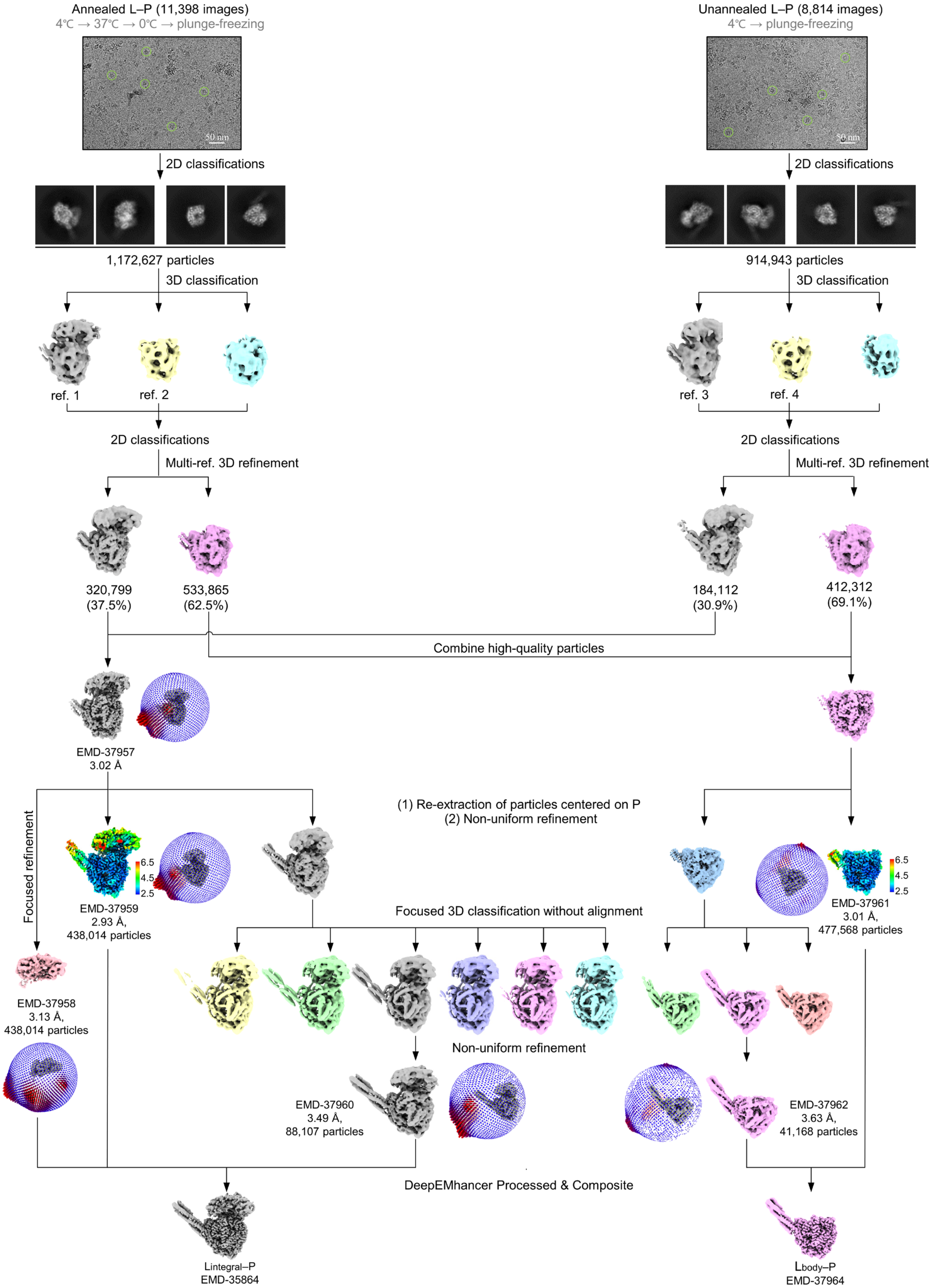

Annealed L–P (11,398 images)
4℃ → 37℃ → 0℃ → plunge-freezing
50 nm
2D classifications
1,172,627 particles
3D classification
ref. 1
ref. 2
2D classifications
Multi-ref. 3D refinement
320,799
(37.5%)
533,865
(62.5%)
Unannealed L–P (8,814 images)
4℃ → plunge-freezing
50 nm
2D classifications
914,943 particles
3D classification
ref. 3
ref. 4
2D classifications
Multi-ref. 3D refinement
184,112
(30.9%)
412,312
(69.1%)
Combine high-quality particles
EMD-37957
3.02 Å
(1) Re-extraction of particles centered on P
(2) Non-uniform refinement
Focused refinement
6.5
4.5
2.5
EMD-37959
2.93 Å,
438,014 particles
EMD-37961
3.01 Å,
477,568 particles
Focused 3D classification without alignment
EMD-37958
3.13 Å,
438,014 particles
Non-uniform refinement
EMD-37960
3.49 Å,
88,107 particles
EMD-37962
3.63 Å,
41,168 particles
DeepEMhancer Processed & Composite
Lintegral–P
EMD-35864
Lbody–P
EMD-37964

Supplementary Fig. 2 Flowchart of cryo-EM data processing on MuV L–P complex. The angular distribution of the particles for the locally refined maps is shown. Local resolution maps for MuV $L_{integral}$–P (EMD-37959) and L_{body}–P (EMD-37961) are shown.

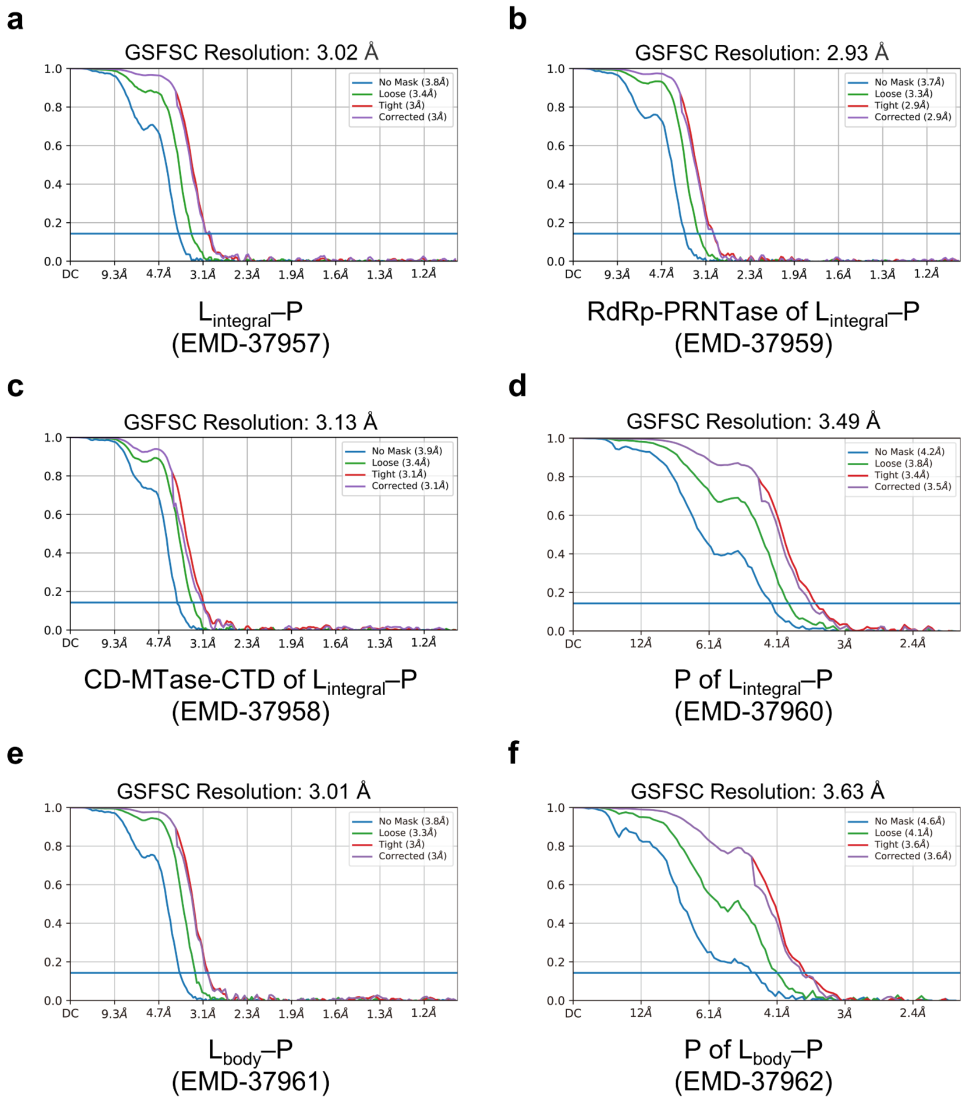

Supplementary Fig. 3 FSC curves of MuV L–P complex. a–d, FSC curves of MuV $L_{integral}$–P. **e,f,** FSC curves of MuV L_{body}–P.

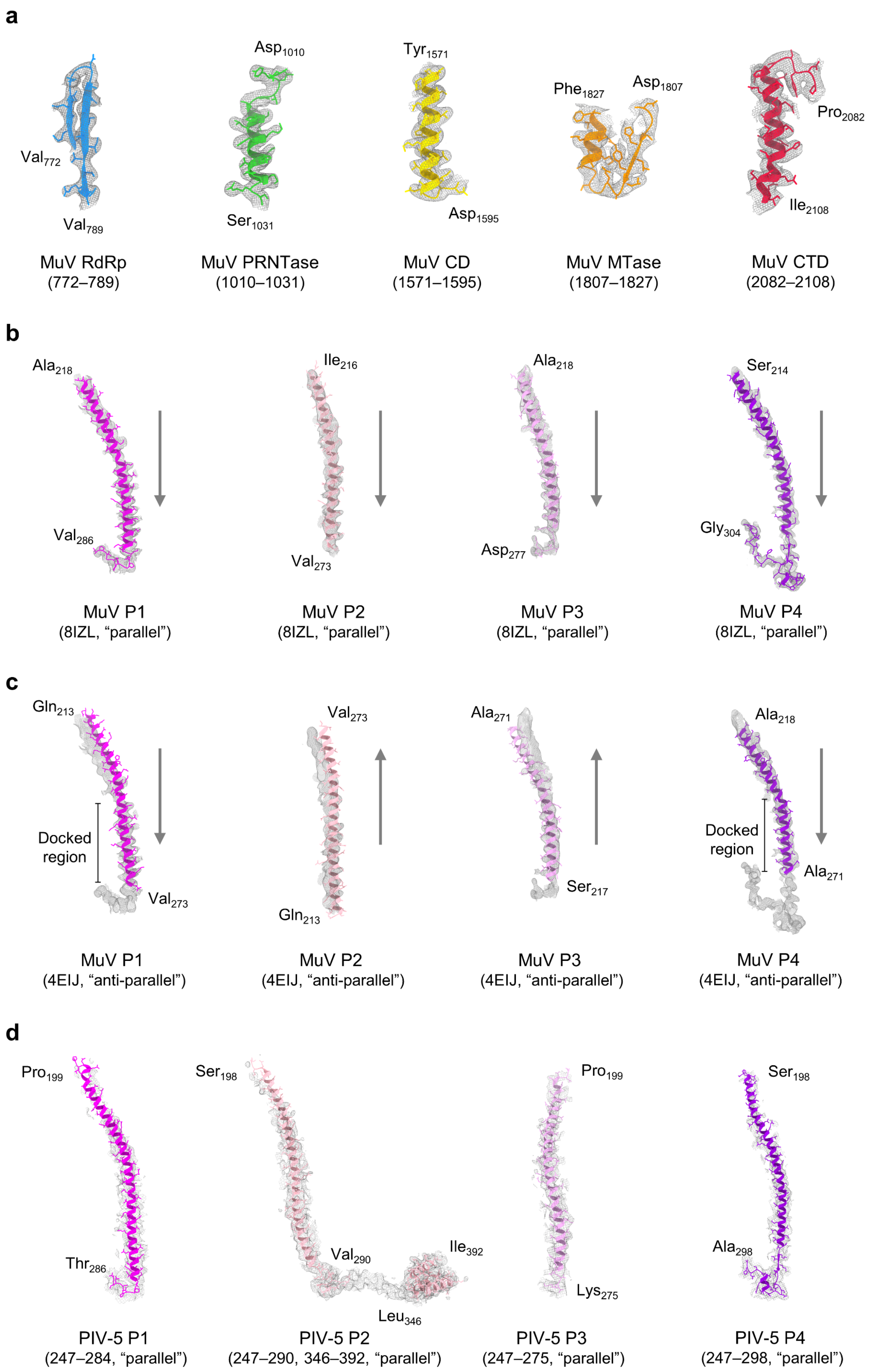

a
Val$_{772}$
Val$_{789}$
MuV RdRp
(772–789)
Asp$_{1010}$
Ser$_{1031}$
MuV PRNTase
(1010–1031)
Tyr$_{1571}$
Asp$_{1595}$
MuV CD
(1571–1595)
Phe$_{1827}$
Asp$_{1807}$
MuV MTase
(1807–1827)
Pro$_{2082}$
Ile$_{2108}$
MuV CTD
(2082–2108)
b
Ala$_{218}$
Val$_{286}$
MuV P1
(8IZL, “parallel”)
Ile$_{216}$
Val$_{273}$
MuV P2
(8IZL, “parallel”)
Ala$_{218}$
Asp$_{277}$
MuV P3
(8IZL, “parallel”)
Ser$_{214}$
Gly$_{304}$
MuV P4
(8IZL, “parallel”)
c
Gln$_{213}$
Docked region
Val$_{273}$
MuV P1
(4EIJ, “anti-parallel”)
Val$_{273}$
Gln$_{213}$
MuV P2
(4EIJ, “anti-parallel”)
Ala$_{271}$
Ser$_{217}$
MuV P3
(4EIJ, “anti-parallel”)
Ala$_{218}$
Docked region
Ala$_{271}$
MuV P4
(4EIJ, “anti-parallel”)
d
Pro$_{199}$
Thr$_{286}$
PIV-5 P1
(247–284, “parallel”)
Ser$_{198}$
Val$_{290}$
Ile$_{392}$
Leu$_{346}$
PIV-5 P2
(247–290, 346–392, “parallel”)
Pro$_{199}$
Lys$_{275}$
PIV-5 P3
(247–275, “parallel”)
Ser$_{198}$
Ala$_{298}$
PIV-5 P4
(247–298, “parallel”)

Supplementary Fig. 4 Representative model fittings into L and P of MuV and PIV-5. a, Representative fragments of MuV L. **b,** Representative fragments of four parallel MuV P molecules (PDB ID 8IZL). Arrow describes the helical direction of each P molecule. **c,** Representative fragments of four antiparallel MuV P molecules (PDB ID 4EIJ). Arrow describes the helical direction of each P molecule. **d,** Representative fragments of PIV-5 P (EMD-21095) and the respective modified atomic models after comparing MuV P with the original model (PDB ID 6V85).

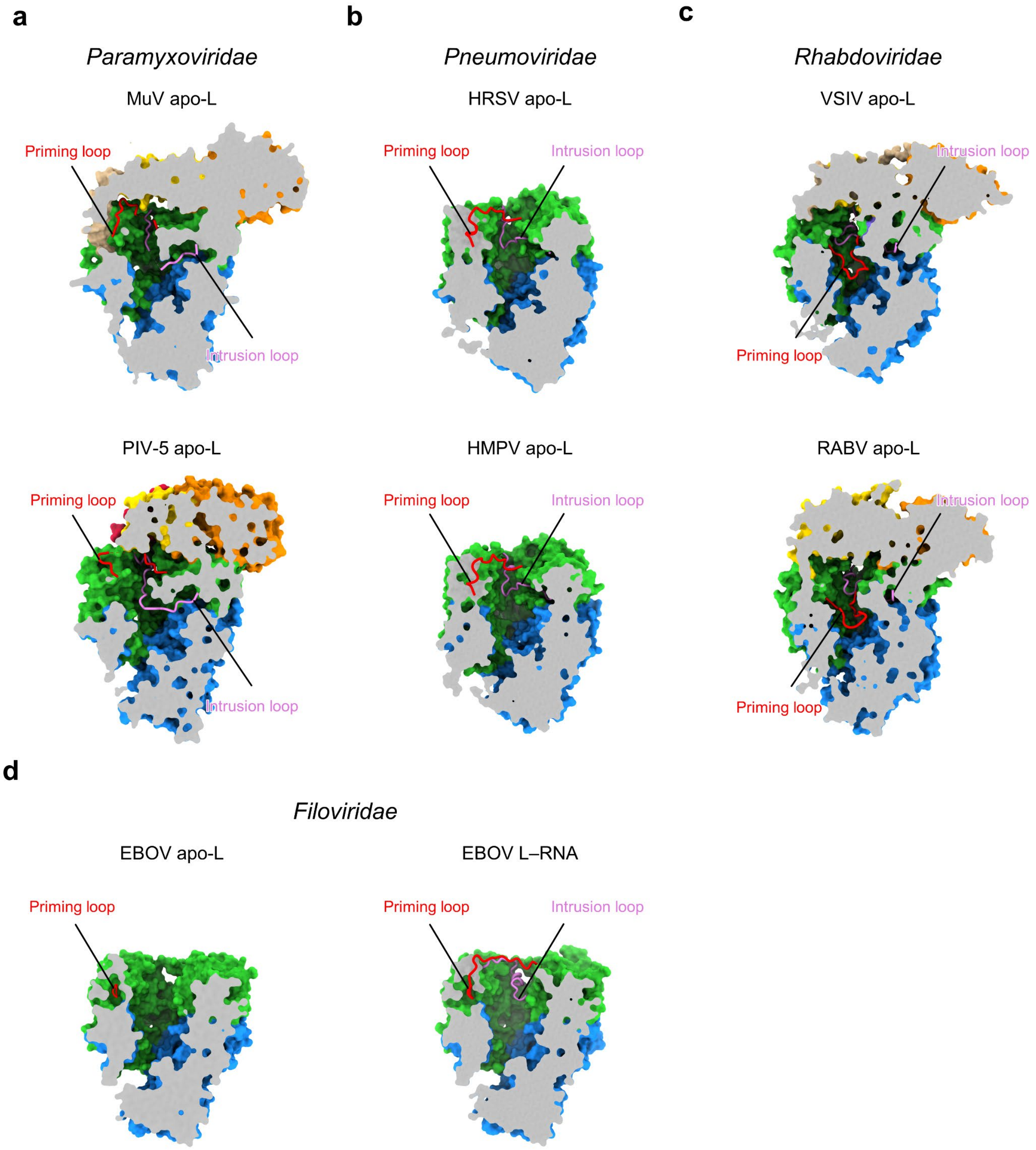

Supplementary Fig. 5 Priming loops and intrusion loops of the structurally resolved nsNSV L. a, The apo-L of MuV and PIV-5 in the family *Paramyxoviridae*. **b,** The apo-L of HRSV and HMPV in the family *Pneumoviridae*. **c,** The apo-L of VSIV and RABV in the family

Rhabdoviridae. **d,** The apo-L and RNA-binding L of EBOV in the family *Filoviridae*. Priming loop, red; Intrusion loop, violet.

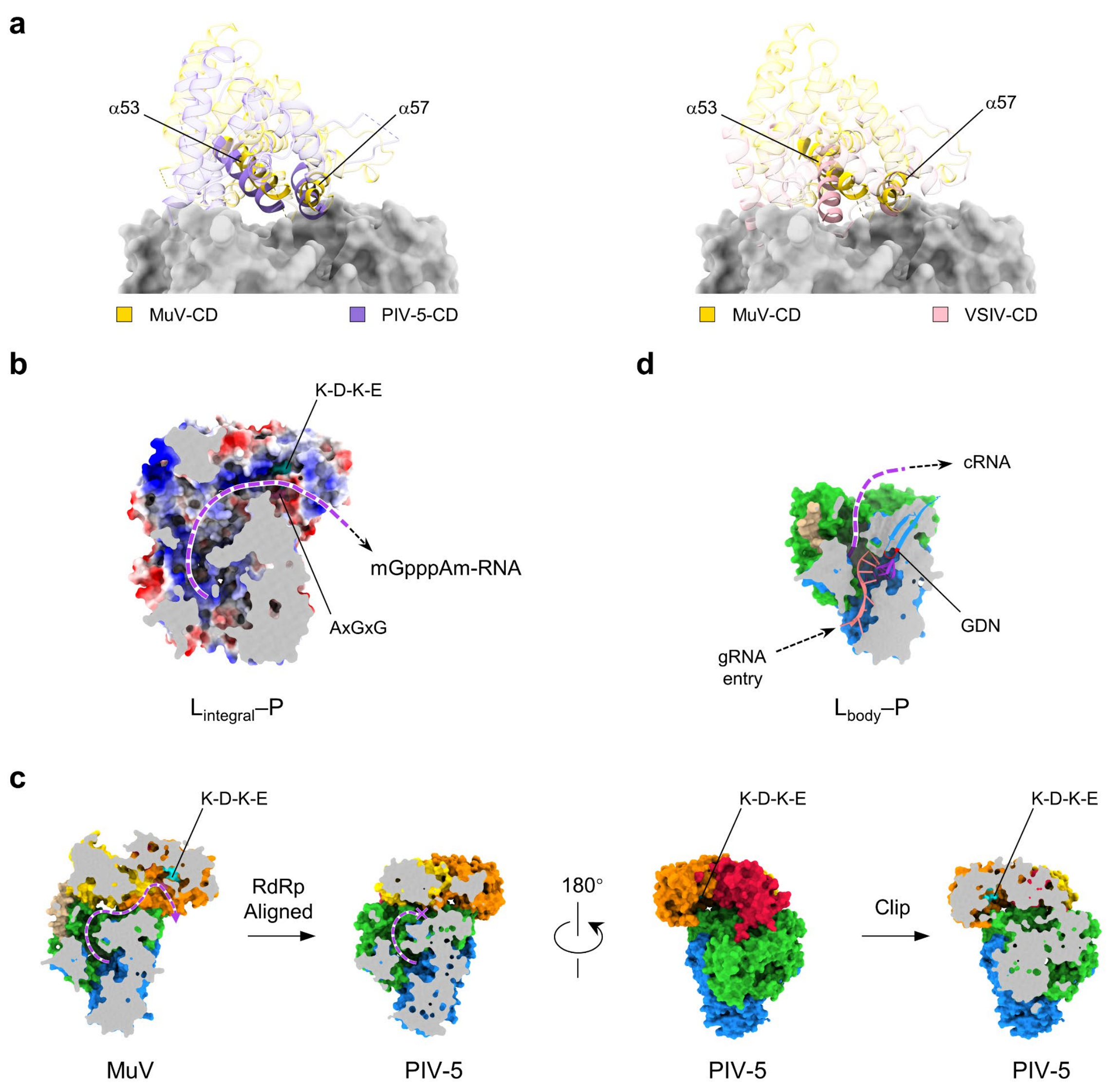

Supplementary Fig. 6 RNA tunnel display. a, Comparison of MuV, PIV-5, and VSIV L CD positioning. Their RdRp and PRNTase are structurally aligned and displayed as grey surfaces. CD of these three viruses are displayed as semi-transparent ribbons. Helices α53 and α57 are highlighted. **b,** Electrostatic surface of MuV L. The purple dashed line indicates the positively charged tunnel for mRNA maturation. The K-D-K-E and AxGxG motifs are colored in cyan and hot pink, respectively. **c,** The K-D-K-E motif of PIV-5 L is not inside the RNA tunnel. **d,** Exposed RNA cavity of MuV L$_{body}$–P.

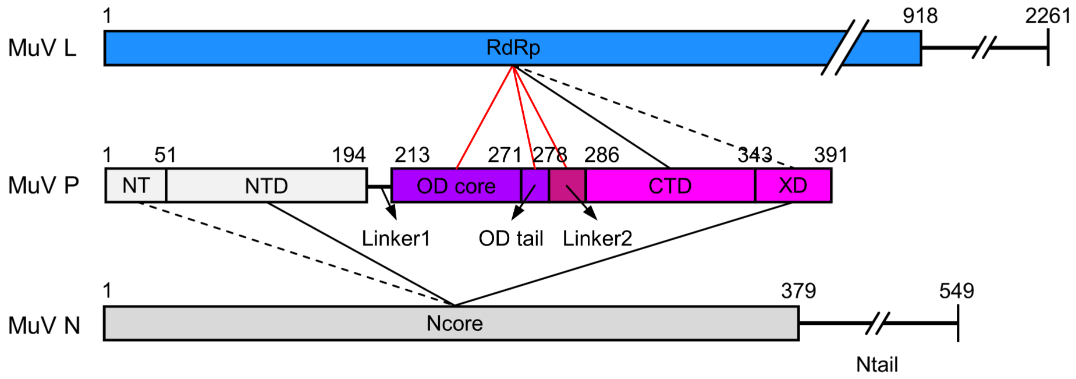

Supplementary Fig. 7 Diagram of the MuV N–P–L interaction map. Red lines: new interactions identified in this work. Black solid lines: interactions proposed by previous studies. Black dashed lines: potential interactions.

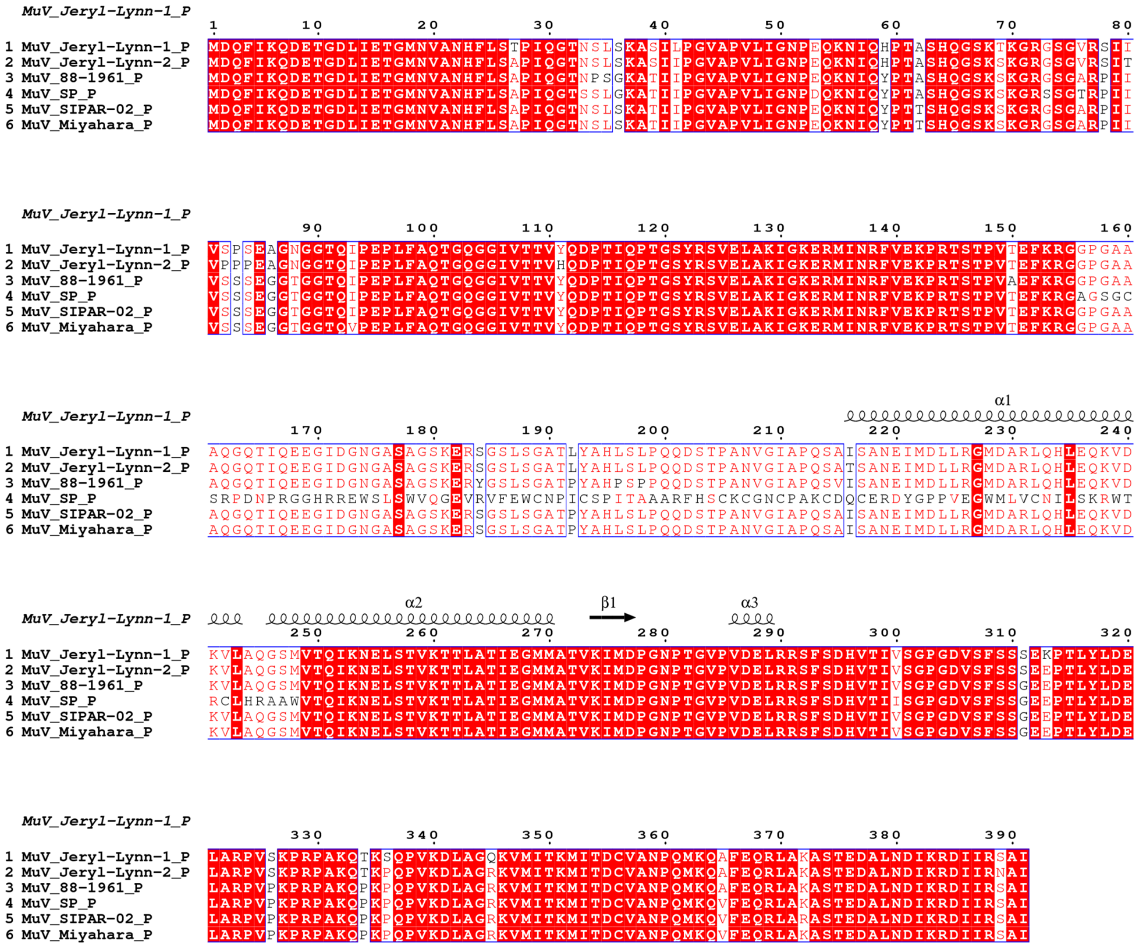

Supplementary Fig. 8 Sequence alignment of P from six MuV strains. Jeryl-Lynn-1, Uniport ID: Q9J4L6; Jeryl-Lynn-2, Uniport ID: Q8QV71; 88-1961, Uniport ID: Q8QY72; SP, Uniport ID: B5TE95; SIPAR-02, Uniport ID: Q910S3; Miyahara, Uniport ID: P30928.

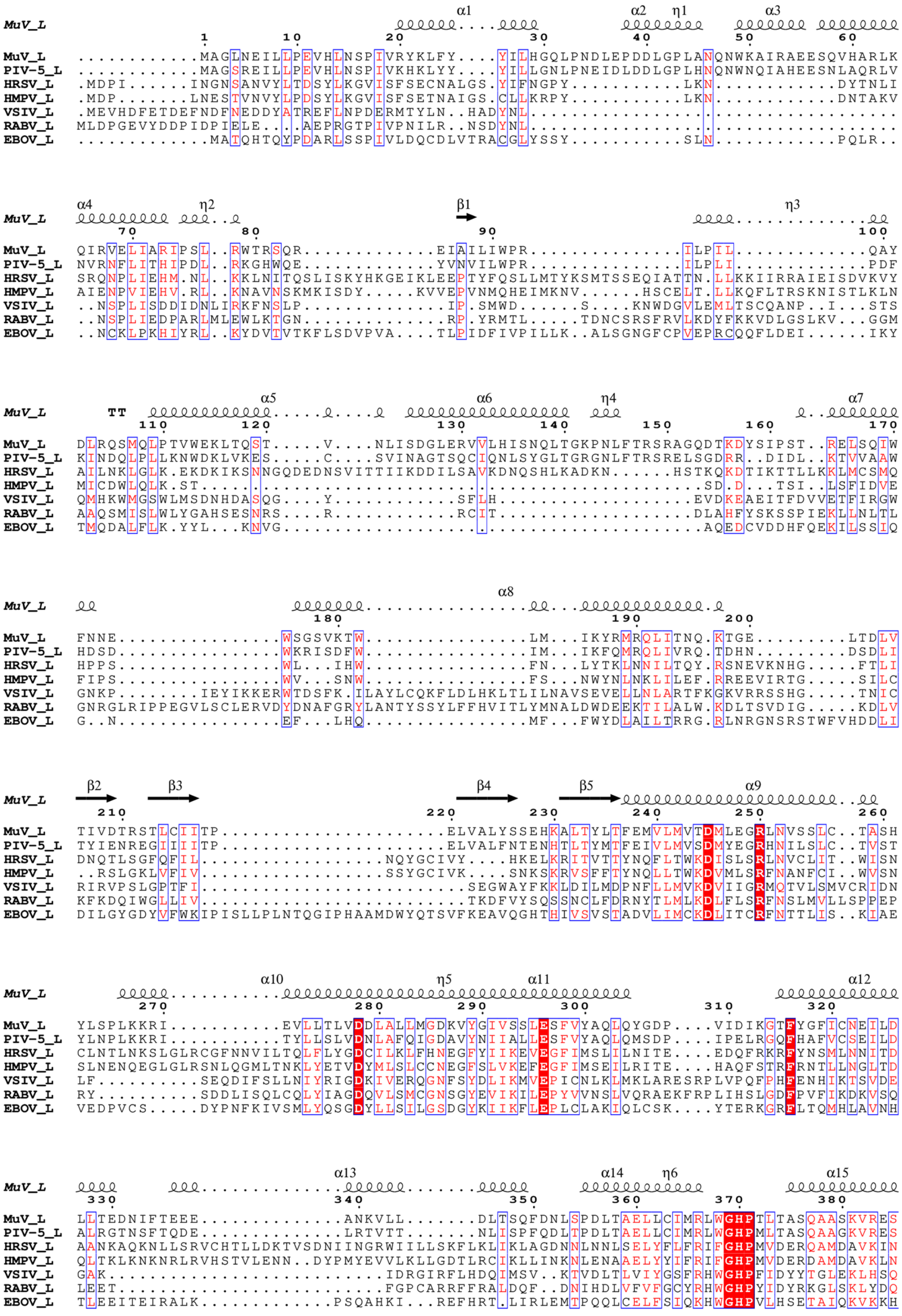

Figure continued on the next page

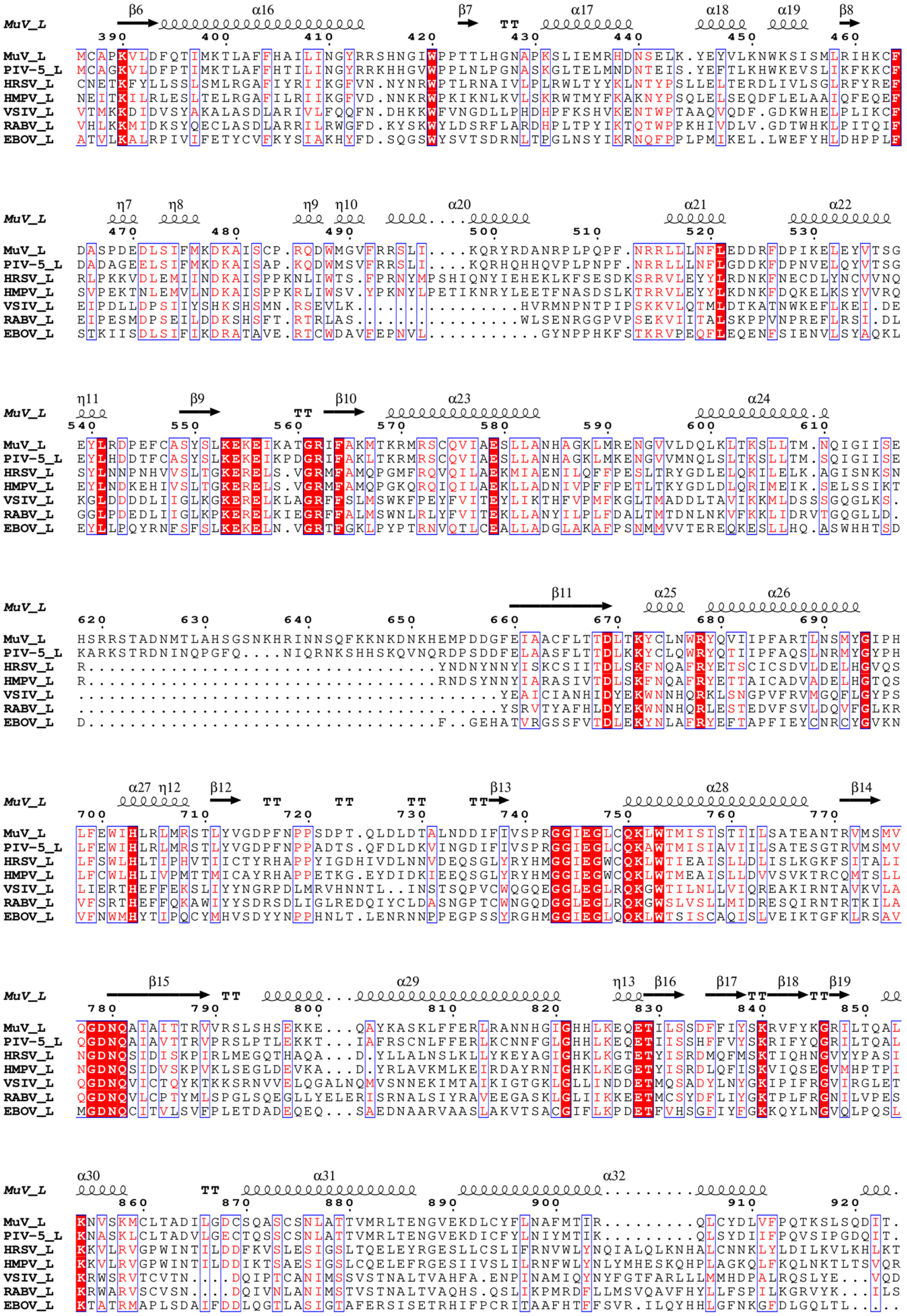

Figure continued on the next page

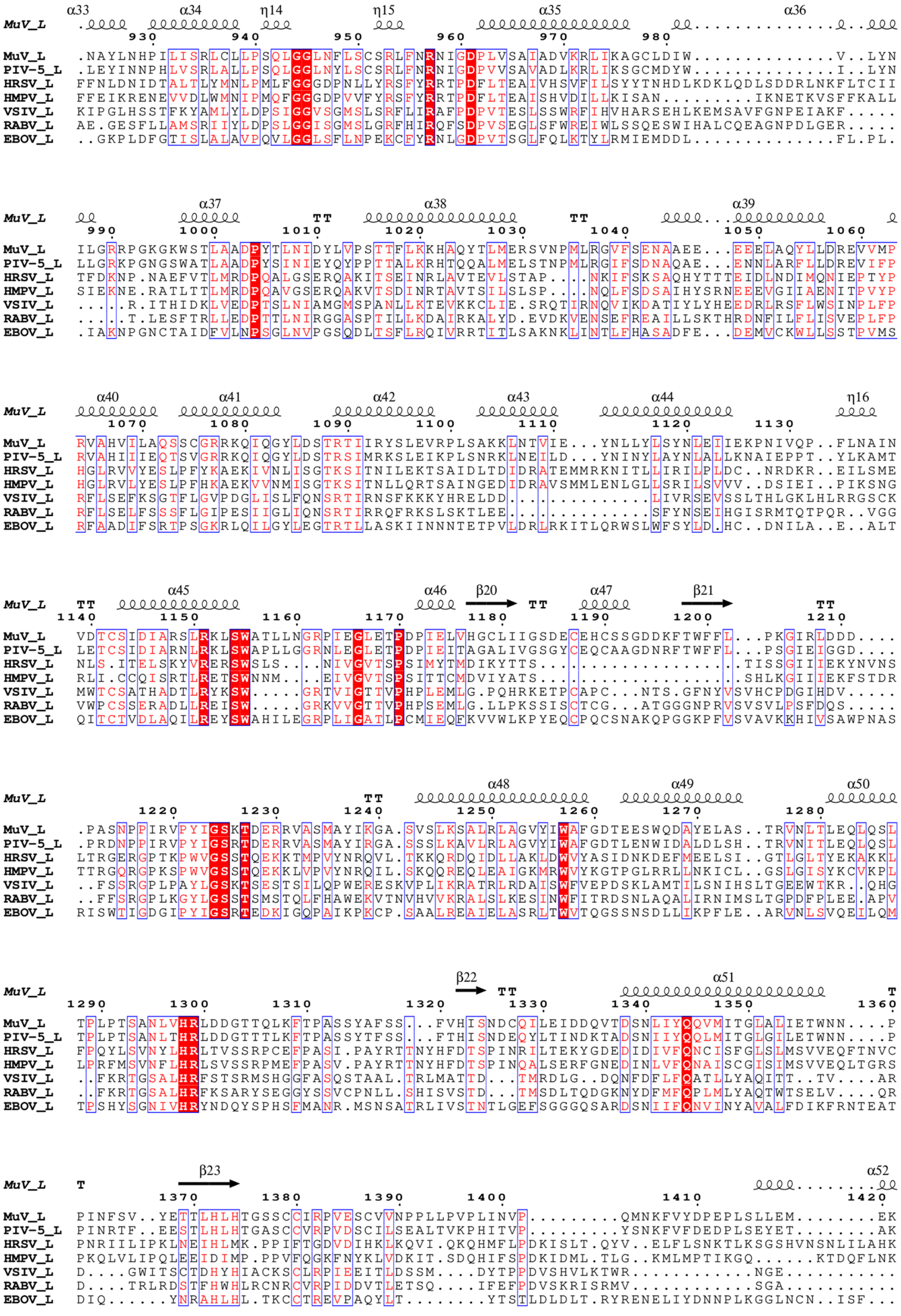

Figure continued on the next page

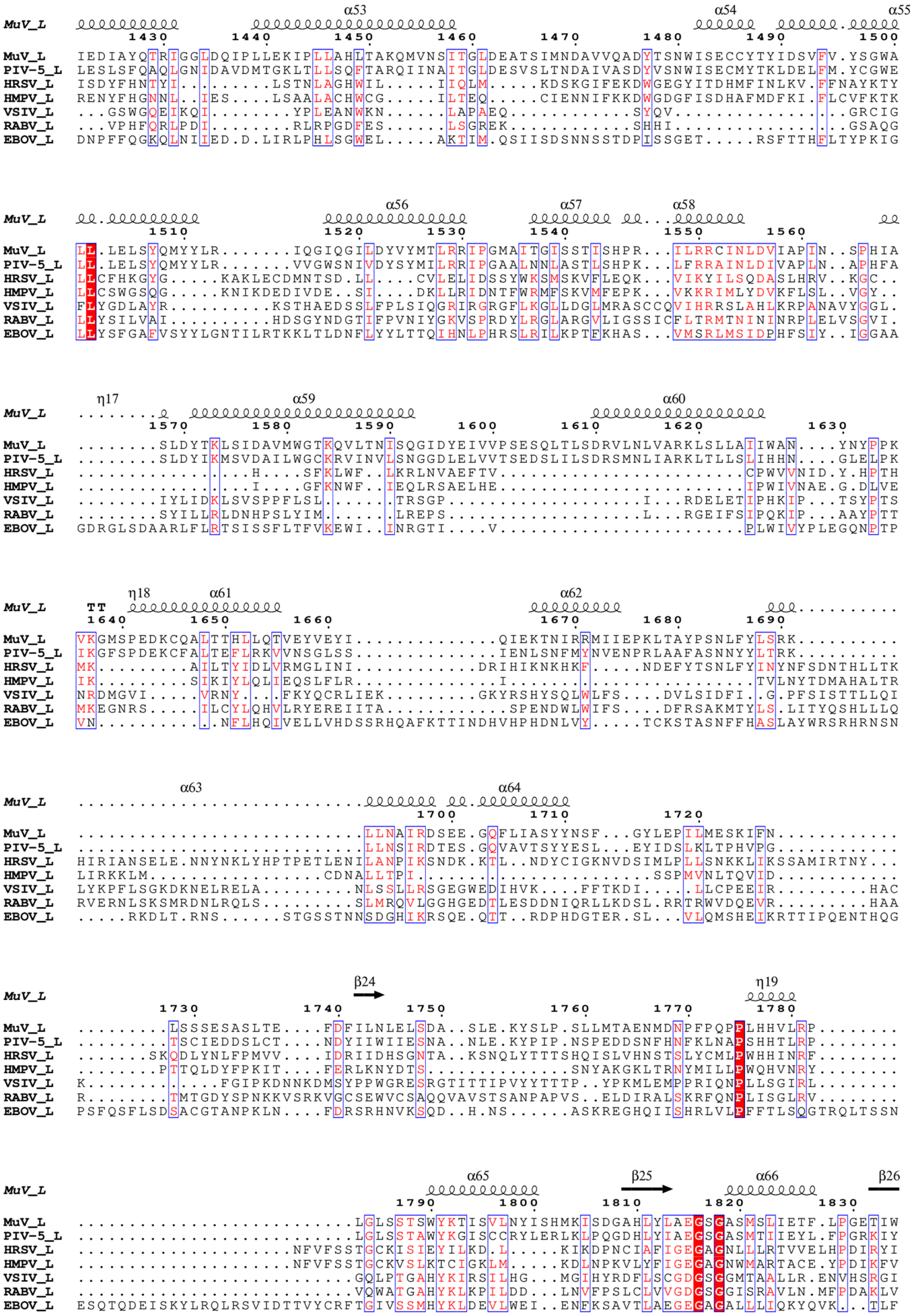

Figure continued on the next page

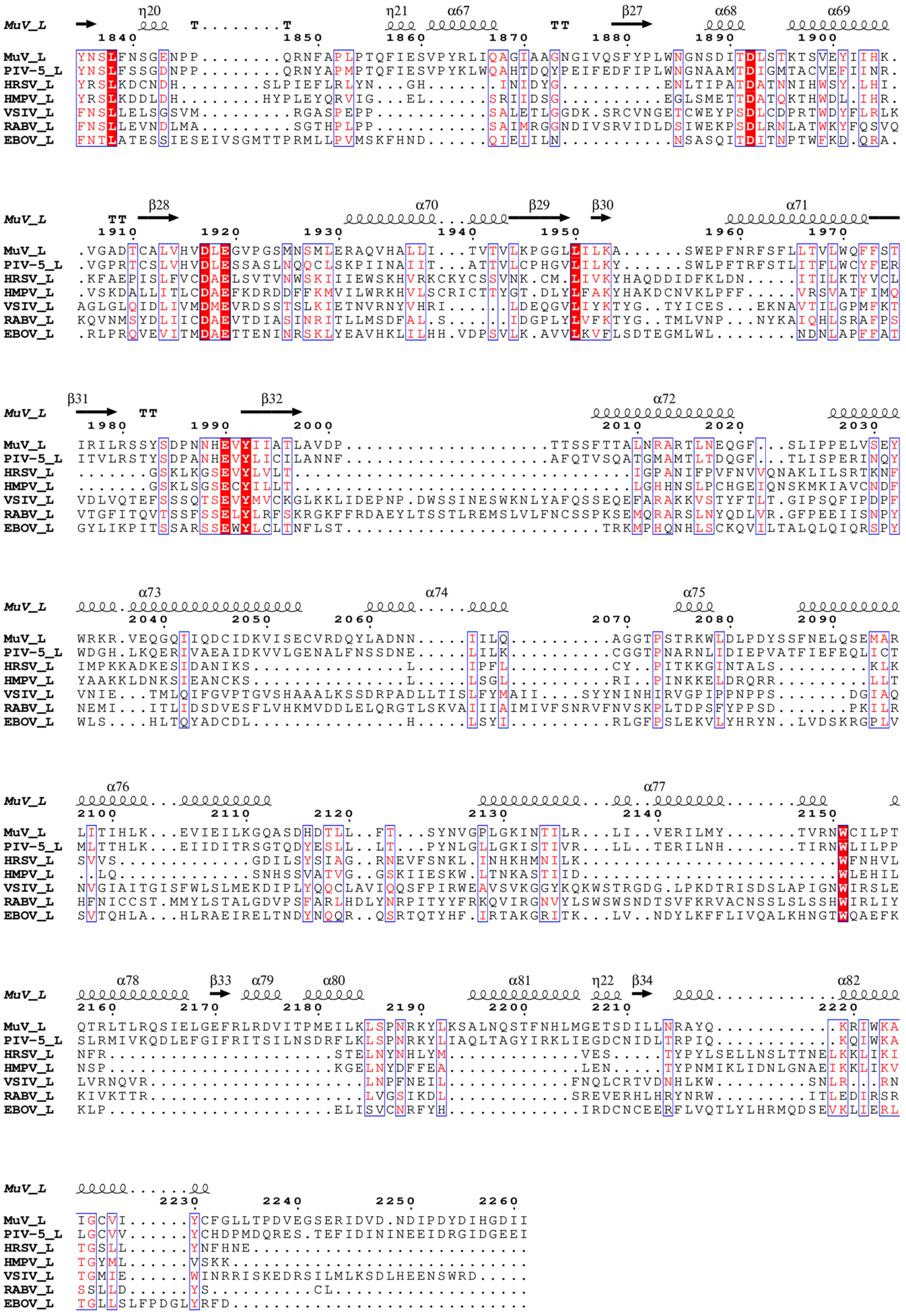

Supplementary Fig. 9 Sequence alignment of nsNSV L. MuV, strain Jeryl-Lynn-1, Uniport ID: Q9J4L0; PIV-5, strain W3, Uniport ID: Q88434; HRSV, strain A2, Uniport ID: P28887; HMPV,

strain CAN97-83, Uniport ID: Q6WB93; VSIV, strain San-Juan, Uniport ID: P03523; RABV, strain SAD-B19, Uniport ID: P16289; EBOV, Uniport ID: X5H5B6.

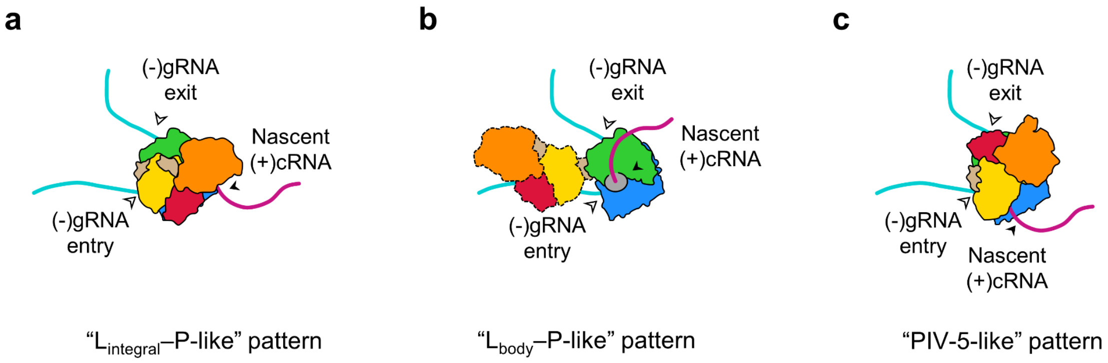

Supplementary Fig. 10 Proposed models for genome replication of nsNSVs. In the replication mode, the conformation of CD-MTase-CTD determines the exit of the complementary RNA (cRNA). **a,** L adopts the “$L_{integral}$–P-like” pattern as in MuV and VSIV. “$L_{integral}$–P-like” structures may bypass the MTase domain for genome replication. **b,** L adopts the “L_{body}–P-like” pattern as in MuV, HRSV, HMPV, and EBOV. The appendage (CD-MTase-CTD) is not fixed on the top of RdRp and PRNTase. The cRNA comes out directly from the central RNA cavity. **c,** L adopts the “PIV-5-like” pattern. The cRNA comes out from the hole fenced by RdRp and CD.

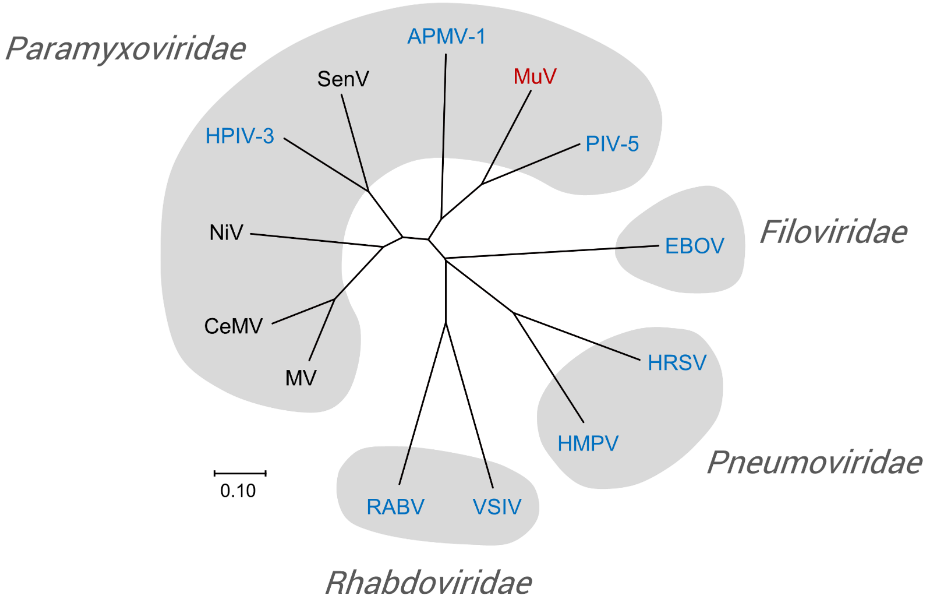

Supplementary Fig. 11 Phylogenetic tree based on L sequences in nsNSVs. Structurally resolved polymerase complexes are labeled in blue and red. MV: measles virus; CeMV: Cetacean morbillivirus; NiV: Nipah virus; HPIV-3: human parainfluenza virus 3; SenV: Sendai virus; APMV-1: Newcastle disease virus; MuV: mumps virus; PIV-5: parainfluenza virus 5; EBOV: Ebola virus; HRSV: human respiratory syncytial virus; HMPV: human metapneumovirus; VSIV: vesicular stomatitis Indiana virus.
