# Supplementary material for: Structures of the mumps virus polymerase complex via cryo-electron microscopy
Source: Nat Commun. 2024 May 17;15:4189. doi: 10.1038/s41467-024-48389-9 (PMC11101452; doi:10.1038/s41467-024-48389-9)
Supplement: Supplementary file 3 — Reporting Summary [file 41467_2024_48389_MOESM3_ESM.pdf]

nature portfolio

Corresponding author(s): Qing-Tao Shen

Last updated by author(s): Apr 3, 2024

Reporting Summary

Nature Portfolio wishes to improve the reproducibility of the work that we publish. This form provides structure for consistency and transparency in reporting. For further information on Nature Portfolio policies, see our Editorial Policies and the Editorial Policy Checklist.

Statistics

For all statistical analyses, confirm that the following items are present in the figure legend, table legend, main text, or Methods section.

n/a	Confirmed	
☐	☒	The exact sample size (*n*) for each experimental group/condition, given as a discrete number and unit of measurement
☒	☐	A statement on whether measurements were taken from distinct samples or whether the same sample was measured repeatedly
☒	☐	The statistical test(s) used AND whether they are one- or two-sided<br>*Only common tests should be described solely by name; describe more complex techniques in the Methods section.*
☒	☐	A description of all covariates tested
☒	☐	A description of any assumptions or corrections, such as tests of normality and adjustment for multiple comparisons
☒	☐	A full description of the statistical parameters including central tendency (e.g. means) or other basic estimates (e.g. regression coefficient) AND variation (e.g. standard deviation) or associated estimates of uncertainty (e.g. confidence intervals)
☒	☐	For null hypothesis testing, the test statistic (e.g. *F*, *t*, *r*) with confidence intervals, effect sizes, degrees of freedom and *P* value noted<br>*Give P values as exact values whenever suitable.*
☒	☐	For Bayesian analysis, information on the choice of priors and Markov chain Monte Carlo settings
☒	☐	For hierarchical and complex designs, identification of the appropriate level for tests and full reporting of outcomes
☒	☐	Estimates of effect sizes (e.g. Cohen's *d*, Pearson's *r*), indicating how they were calculated

Our web collection on statistics for biologists contains articles on many of the points above.

Software and code

Policy information about availability of computer code

Data collection	FEI EPU (Thermo Fisher Scientific) was used for cryo-EM data collection.
Data analysis	MotionCor2.1, CTFFIND4, CryoSPARC v3.1, DeepEMhancer, SWISS-MODEL, RoseTTAFold, UCSF ChimeraX 1.5, COOT 0.9.7, PHENIX 1.20.1, MolProbity 4.3.1, PDBePISA 1.48, Clustal Omega, ESPript 3.0, MEGA 11.

For manuscripts utilizing custom algorithms or software that are central to the research but not yet described in published literature, software must be made available to editors and reviewers. We strongly encourage code deposition in a community repository (e.g. GitHub). See the Nature Portfolio guidelines for submitting code & software for further information.

Data

Policy information about availability of data

All manuscripts must include a data availability statement. This statement should provide the following information, where applicable:

- Accession codes, unique identifiers, or web links for publicly available datasets
- A description of any restrictions on data availability
- For clinical datasets or third party data, please ensure that the statement adheres to our policy

The cryo-EM density maps, including the globally refined maps, locally refined maps, and the DeepEMhancer processed composite maps, have been deposited to the Electron Microscopy Data Bank (EMDB, https://www.ebi.ac.uk/pdbe/emdb/). The atomic coordinates corresponding to the locally refined maps and the composite maps of MuV Lintegral–P and Lbody–P have been deposited to the Protein Data Bank (PDB, https://www.rcsb.org/). The accession numbers are listed as

follows: EMD-37957 (Lintegral–P as the whole), EMD-37959 and PDB ID 8YXM (RdRp-PRNTase of Lintegral–P), EMD-37958 and PDB ID 8YXL (CD-MTase-CTD of Lintegral–P), EMD-37960 and PDB ID 8YXO (P of Lintegral–P), and EMD-35864 and PDB ID 8IZL (the composite map of Lintegral–P from EMD-37959, EMD-37958, and EMD-37960 and the composite model from PDB IDs 8YXM, 8YXL and 8YXO); EMD-37961 and PDB ID 8YXP (Lbody–P as the whole), EMD-37962 and PDB ID 8YXR (P of Lbody–P), and EMD-37964 and PDB ID 8X01 (the composite map of Lbody–P from EMD-37961 and EMD-37962 and the composite model from PDB IDs 8YXP and 8YXR).

Research involving human participants, their data, or biological material

Policy information about studies with human participants or human data. See also policy information about sex, gender (identity/presentation), and sexual orientation and race, ethnicity and racism.

Reporting on sex and gender	n/a
Reporting on race, ethnicity, or other socially relevant groupings	n/a
Population characteristics	n/a
Recruitment	n/a
Ethics oversight	n/a

Note that full information on the approval of the study protocol must also be provided in the manuscript.

Field-specific reporting

Please select the one below that is the best fit for your research. If you are not sure, read the appropriate sections before making your selection.

☒ Life sciences ☐ Behavioural & social sciences ☐ Ecological, evolutionary & environmental sciences

For a reference copy of the document with all sections, see nature.com/documents/nr-reporting-summary-flat.pdf

Life sciences study design

All studies must disclose on these points even when the disclosure is negative.

Sample size	11,398 and 8,814 micrographs were collected for annealed and unannealed MuV L–P samples, respectively. Accordingly, 1,172,627 particles (the annealing group) and 914,943 particles (the unannealed group) were selected from micrographs. No statistical methods were used to predetermine sample size.
Data exclusions	317,963 particles (the annealing group) and 318,519 particles (the unannealed group) were excluded as obvious junks after two-dimensional classification.
Replication	All biochemical experiments including the de novo RNA synthesis assay and the primer-extension assay were performed at least three times. All replicas of data produced similar results.
Randomization	This is not relevant to this study, because no grouping was needed.
Blinding	Investigators were not blinded to group allocation, because no grouping was needed for this study.

Reporting for specific materials, systems and methods

We require information from authors about some types of materials, experimental systems and methods used in many studies. Here, indicate whether each material, system or method listed is relevant to your study. If you are not sure if a list item applies to your research, read the appropriate section before selecting a response.

Materials & experimental systems

n/a	Involved in the study
☐	☒ Antibodies
☐	☒ Eukaryotic cell lines
☒	☐ Palaeontology and archaeology
☒	☐ Animals and other organisms
☒	☐ Clinical data
☒	☐ Dual use research of concern
☒	☐ Plants

Methods

n/a	Involved in the study
☒	☐ ChIP-seq
☒	☐ Flow cytometry
☒	☐ MRI-based neuroimaging

Antibodies

Antibodies used: The horse anti-Strep (IBA Lifesciences, catalog number 2-1502-001) monoclonal antibody was used to detect L proteins at the dilution of 1:100,000, and the mouse anti-Flag (Sigma, catalog number F1804) monoclonal antibody were used to detect P proteins at the dilution of 1:1,000 in western blot analyses.

Validation: The horse anti-Strep and mouse anti-Flag monoclonal antibodies have been validated by the manufacture. The validation materials can be found on the companies' website.

Eukaryotic cell lines

Policy information about cell lines and Sex and Gender in Research

Cell line source(s): Insect cell line Sf9, purchased from the Invitrogen company, were used for protein expression.

Authentication: These cells are routinely maintained in our lab. No other authentication at the lab level was performed.

Mycoplasma contamination: All cells were tested negative for Mycoplasma contamination.

Commonly misidentified lines (See ICLAC register): No commonly misidentified cells were used in this study.
